# Association of neighborhood gentrification with prostate cancer and immune markers in African American and European American men

**DOI:** 10.1002/cam4.6828

**Published:** 2023-12-27

**Authors:** Catherine M. Pichardo, Adaora Ezeani, Margaret S. Pichardo, Tanya Agurs‐Collins, Tiffany M. Powell‐Wiley, Brid Ryan, Tsion Zewdu Minas, Maeve Bailey‐Whyte, Wei Tang, Tiffany H. Dorsey, William Wooten, Christopher A. Loffredo, Stefan Ambs

**Affiliations:** ^1^ Division of Cancer Control and Population Sciences, NCI NIH Rockville Maryland USA; ^2^ Department of Surgery, Hospital of the University of Pennsylvania Penn Medicine Philadelphia Pennsylvania USA; ^3^ Social Determinants of Obesity and Cardiovascular Risk Laboratory, Cardiovascular Branch, Division of Intramural Research, National Heart, Lung, and Blood Institute (NHLBI) National Institutes of Health Bethesda Maryland USA; ^4^ Intramural Research Program, National Institute on Minority Health and Health Disparities (NIMHD) National Institutes of Health Bethesda Maryland USA; ^5^ Laboratory of Human Carcinogenesis, National Cancer Institute (NCI) National Institutes of Health (NIH) Bethesda Maryland USA; ^6^ School of Medicine University of Limerick Limerick Ireland; ^7^ Data Science & Artificial Intelligence, R&D AstraZeneca Gaithersburg Maryland USA; ^8^ University of Maryland Marlene and Stewart Greenebaum Comprehensive Cancer Center Biostatistics Shared Service Baltimore Maryland USA; ^9^ Cancer Prevention and Control Program, Lombardi Comprehensive Cancer Center Georgetown University Medical Center Washington District of Columbia USA

**Keywords:** health disparity, immune markers, minority and vulnerable populations, mortality, neighborhood, prostate cancer

## Abstract

**Background:**

Prior studies showed that neighborhood deprivation increases the risk of lethal prostate cancer. However, the role of neighborhood gentrification in prostate cancer development and outcome remains poorly understood. We examined the relationships of gentrification with prostate cancer and serum proteome‐defined inflammation and immune function in a diverse cohort.

**Methods:**

The case–control study included 769 cases [405 African American (AA), 364 European American (EA) men] and 1023 controls (479 AA and 544 EA), with 219 all‐cause and 59 prostate cancer‐specific deaths among cases. Geocodes were linked to a neighborhood gentrification index (NGI) derived from US Census data. Cox and logistic regression, and MANOVA, were used to determine associations between NGI, as continuous or quintiles (Q), and outcomes.

**Results:**

Adjusting for individual socioeconomic status (SES), continuous NGI was positively associated with prostate cancer among all men (odds ratio [OR] 1.07, 95% confidence interval [CI] 1.01–1.14). AA and low‐income men experienced the highest odds of prostate cancer when residing in tracts with moderate gentrification, whereas EA men experienced reduced odds of regional/metastatic cancer with increased gentrification in SES‐adjusted analyses. Continuous NGI also associated with mortality among men presenting with localized disease and low‐income men in SES‐adjusted Cox regression analyses. NGI was not associated with serum proteome‐defined chemotaxis, inflammation, and tumor immunity suppression.

**Conclusions:**

Findings show that neighborhood gentrification associates with prostate cancer and mortality in this diverse population albeit associations were heterogenous within subgroups. The observations suggest that changing neighborhood socioeconomic environments may affect prostate cancer risk and outcome, likely through multifactorial mechanisms.

## 1 INTRODUCTION

African American (AA) men experience the highest rates of lethal prostate cancer compared to other groups in the United States.^1^ Adverse neighborhood environments contribute to cancer inequities in part because they control access to health‐promoting resources.^2^, ^3^, ^4^, ^5^, ^6^, ^7^, ^8^ In addition, adverse social neighborhood conditions can directly or indirectly influence prostate cancer outcomes by inducing chronic stress‐related biological responses, oxidative stress, and chronic inflammation.^2^, ^7^

Neighborhoods are rapidly changing and characterizing the effects that these changing environments, particularly neighborhood gentrification, may have on cancer disparities is of significance, and remains poorly understood.^9^, ^10^, ^11^ Neighborhood gentrification (herein referred to as gentrification) is defined as a “process of neighborhood change through which the demographic, real estate, and business characteristics of a place transition toward a population that is wealthier, Whiter, has a higher level of formal education, and is able to afford new or renovated, more expensive homes while also fomenting new cultural and consumption practices.”^12^ That is, gentrification is an urban restructuring process that involves socioeconomic upgrading of originally low‐income neighborhoods, infrastructure development, real estate enhancement, and rising home values and rent.^13^, ^14^ Tied to gentrification‐related neighborhood changes are resegregation and displacement of low‐income residents who are healthy to move,^15^, ^16^ as well as displacement of health protective social networks, social capital, health promoting community institutions, and medical resources tailored for underserved groups.^12^, ^14^, ^17^, ^18^ Although studies of the neighborhood environment have provided evidence that neighborhood deprivation increases the risk of lethal prostate cancer,^7^ the impacts of gentrification on prostate cancer outcomes is not well researched and understood.^6^, ^17^

Therefore, using a large case–control study of AA and European American (EA) men, we examined the association of gentrification between the Decennial Census 1990 and the 2006–2010 American Community Survey with prostate cancer, disease mortality, and systemic inflammation and immune function.

## 2 METHODS

The NCI‐Maryland Prostate Cancer Case–Control Study recruited 976 cases (489 AA and 487 EA men) and 1034 population controls (486 EA and 548 AA men) from 2005 to 2015 aged between 40 and 90 years. Details of the study and eligibility criteria have been published elsewhere.^19^, ^20^ Participants were recruited from the greater Baltimore area in Maryland frequency matched by age and race. Participants completed a series of questionnaires and blood draws. Baseline addresses were geocoded using 2010 US Census Tracts Federal Information Processing Series boundaries, and were linked to both census data from the 1990 Decennial Census and the 2006–2010 American Community Survey (ACS) estimates derived from the National Neighborhood Change Database.^21^ Individuals with missing baseline home addresses (*n* = 86), prevalent cases (i.e., recruitment >1 year post disease diagnosis) (*n* = 131), and missing body mass index (*n* = 1) were excluded, yielding an analytic sample of 1792 men: 769 cases (405 AA and 364 EA) and 1023 controls (479 AA and 544 EA), with 219 all‐cause (122 AA and 97 EA) and 59 prostate cancer‐specific (36 AA and 23 EA) deaths among cases.

### 2.1 Neighborhood gentrification

An index of census tract gentrification from the Decennial Census 1990 to ACS 2006–2010 was constructed based on prior work in the United States.^22^, ^23^, ^24^ The index represents the sum of z scores of percent change of three socioeconomic indicators (i.e., college or more educated adults aged 25 or more, number of residents living below the federal poverty line, and median household income). Greater scores indicate greater gentrification (increases in neighborhood socioeconomic status). The gentrification variable was analyzed as continuous or quintiles (Q). The means and ranges for the gentrification index quintiles are as follows: Q1 mean = −2.33, range: −7.94 to −1.34; Q2 mean = −0.84, range: −1.33 to −0.44; Q3 mean = −0.16, range: −0.44 to 0.11; Q4 mean = 0.45, range: 0.12 to 0.76; Q3 mean = 1.76, range: 0.76 to 19.16.

### 2.2 Prostate cancer risk and outcomes

Incident prostate cancer was defined as being enrolled in the study within 12 months from diagnosis, with an average interval of 4.8 months between diagnosis and enrollment. Exclusion of those with recruitment >1 year post‐disease diagnosis reduces selection bias as participants diagnosed within a year of recruitment may have different disease characteristics. Death surveillance occurred through December 31, 2020 and causes of death were drawn from the National Death Index. Among cases, survival was calculated from date of diagnosis to either date of death or censor date of December 31, 2020. Death from other causes than prostate cancer was identified as a competing event.

### 2.3 Serum proteomics‐defined biological pathways

Pathway/biological process activity scores were derived from 82 circulating serum protein measurements. Levels of these proteins were assessed using the proprietary multiplex Proximal Extension Assay by Olink Proteomics (Boston), as previously reported by us.^25^ Following Olink guidelines, proteins were grouped into six pathways. To create pathway/biological process activity scores, *z*‐scores for each of the serum proteins in a pathway were calculated that reflect their abundance and summed up into one score for each participant, creating a participant's pathway activity score as described.^25^ All protein markers included in this analysis passed a stringent quality control measure with their coefficients of variation (CV) among blinded duplicates being <10%. We then examined associations between neighborhood gentrification and either suppression of tumor immunity (Th2 response), chemotaxis/trafficking to tumor, or inflammation scores, as these were previously linked to neighborhood deprivation for this cohort.^7^

### 2.4 Statistical analysis

We used logistic regression models to examine the association of gentrification with prostate cancer. To analyze the association between gentrification and National Comprehensive Cancer Network (NCCN)‐defined risk scores for prostate cancer, we categorized cases into the following risk categories: (1) low risk (referent), (2) intermediate risk, (3) high or very high risk, and (4) regional or metastatic disease.

Multivariable Cox proportional hazard regression models [hazard ratios (HR) or cause‐specific hazard ratio (CSHR), and 95% CI] were used to determine all‐cause and prostate cancer‐specific mortality among cases. We also fitted the Fine‐Gray subdistribution hazard (SHR) model for prostate cancer‐specific mortality, to account for competing risks (i.e., death from other cancers or causes). Multivariate analysis of variance (MANOVA) models assessed the relationship between gentrification and activity scores of six biological process/pathways based on 82 immuno‐oncology markers. Wilk's lambda (referred to as the *U*‐statistic) was used to determine significant effects of gentrification. This analysis was performed on the control population only to exclude the effect of prostate cancer. Our analyses were conducted for the overall study population and stratified by self‐reported race (AA and EA) or income (low‐income men defined as <$30,000 and middle‐/high‐income men as ≥$30,000). Covariates included in analysis are described in Supplementary Methods. All analyses were conducted in Stata 17.0 and statistical significance was defined as *p* < 0.05. Statistical significance in pathways was accepted at the Bonferroni‐adjusted *p* < 0.05.

Additional information on the study population, including serum protein measurement and derived biological process/pathways, functional annotation and biological process score system, classification of cases using NCCN risk scores, and West African ancestry estimation has been published elsewhere.^7^

## 3 RESULTS

### 3.1 Sample characteristics

Tables S1 and S2 show participants' characteristics stratified by gentrification quintiles for all men combined and separately for AA and EA men, as well as mean gentrification scores by participant characteristics. Per study design, controls were matched to cases on age (5‐year intervals), race/ethnicity, and residency. Despite these matching criteria, AA cases had a lower education level than the controls. A clustering pattern analysis showed that cases were widely dispersed across the census tracts in which these men resided, as previously reported.^7^ Education was associated with gentrification among both EA and AA controls, whereas income associated with gentrification only among AA controls and cases.

### 3.2 Association of neighborhood gentrification with prostate cancer

**FIGURE 1:**
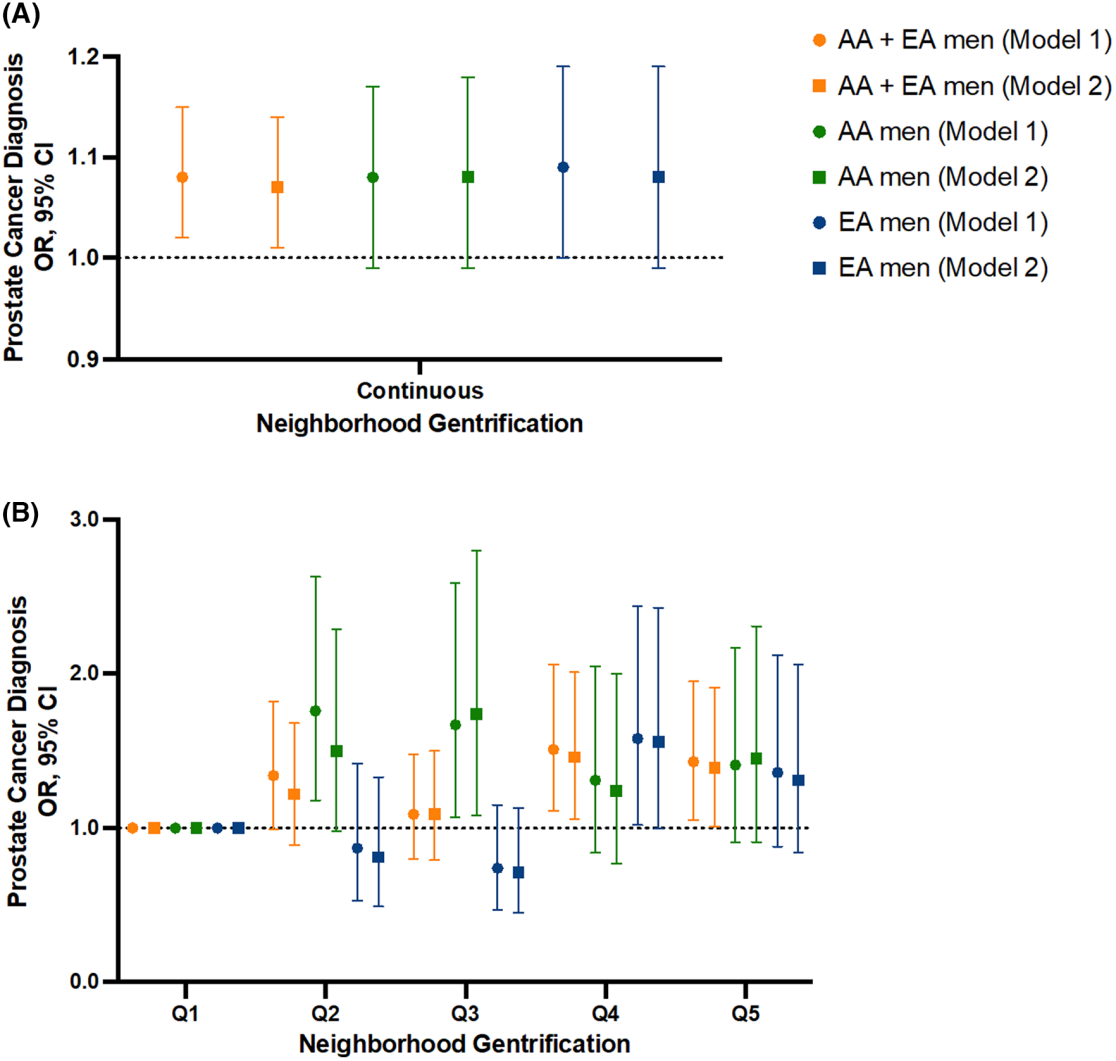
Association of neighborhood gentrification with a prostate cancer diagnosis among African American (AA) and European American (EA) men. Odds ratios (OR) and 95% confidence interval (CI) for a prostate cancer diagnosis by neighborhood gentrification with gentrification coded as (A) continuous score and (B) quintiles, with additional stratification by self‐reported race/ethnicity. Logistic regression with Model 1 being adjusted for all baseline covariates, whereas Model 2 adjusted for all covariates with the additional inclusion of education and individual income.

Multivariable logistic regression analysis revealed an association of gentrification with prostate cancer among all men when either continuous data or quintiles were used in the models (see Table 1; Figure 1). High gentrification (Q4‐5) was associated with 43%–50% increased odds of prostate cancer. The association remained significant with additional SES adjustment (Q4 vs. Q1 OR model 2: 1.45, 95% CI: 1.06–2.00; Q5 vs. Q1 OR model 2: 1.39, 95% CI: 1.01–1.91). In SES‐adjusted models stratified by race/ethnicity, AA men experienced the highest odds of prostate cancer when residing within tracts with moderate (Q3) levels of gentrification (Table 1; Figure 1B). We did not observe the same association with EA men. Among models of all men combined and EA men only, there was a suggestive positive trend between increased gentrification and increased odds of prostate cancer, with and without SES adjustment (*p* trend <0.05 across quintiles) (Table 1). When we stratified men by income, gentrification was associated with increased odds of prostate cancer in the low‐income group, with or without further adjustment for education (Table 1). In this analysis, low‐income men experienced the highest odds of prostate cancer when residing in tracts with moderate gentrification. No association was found among middle‐ and high‐income men.

**TABLE 1:** Association between neighborhood gentrification and a prostate cancer diagnosis in the NCI‐Maryland Prostate Cancer Case–Control Study.

	AA + EA men		AA men		EA men		Low‐income men		Middle‐/high‐income men	
	Model 1, OR (95% CI)	Model 2, OR (95% CI)	Model 1, OR (95% CI)	Model 2, OR (95% CI)	Model 1, OR (95% CI)	Model 2, OR (95% CI)	Model 1, OR (95% CI)	Model 2, OR (95% CI)	Model 1, OR (95% CI)	Model 1, OR (95% CI)
	*N* = 1792	*N* = 1792	*N* = 884	*N* = 884	*N* = 908	*N* = 908	*N* = 453	*N* = 453	*N* = 1339	*N* = 1339
Neighborhood gentrification 1990–2010										
Continuous, OR [95% CI]	**1.08** * **[1.02, 1.15]**	**1.07** * **[1.01, 1.14]**	1.08 [0.99, 1.17]	1.08 [0.99, 1.18]	1.09 [1.00, 1.19]	1.08 [0.99, 1.19]	**1.28** ** **[1.10, 1.48]**	**1.27** ** **[1.10, 1.47]**	1.03 [0.96, 1.11]	1.03 [0.96, 1.10]
Quintile, OR [95% CI]										
Q1 (very low)	Ref.	Ref.	Ref.	Ref.	Ref.	Ref.				
Q2	1.34 [0.99, 1.82]	1.22 [0.89, 1.67]	**1.79** ** **[1.20, 2.66]**	1.52 [1.00, 2.32]	0.85 [0.52, 1.39]	0.79 [0.48, 1.31]	1.79 [0.98, 3.27]	1.77 [0.97, 3.25]	1.20 [0.84, 1.73]	1.17 [0.81, 1.68]
Q3	1.09 [0.80, 1.49]	1.09 [0.79, 1.50]	**1.70** * **[1.09, 2.64]**	**1.79** * **[1.11, 2.88]**	0.72 [0.46, 1.13]	0.70 [0.44, 1.10]	**2.13** * **[1.10, 4.12]**	**2.18** * **[1.12, 4.25]**	0.85 [0.59, 1.22]	0.87 [0.61, 1.25]
Q4	**1.50** ** **[1.10, 2.04]**	**1.45** * **[1.06, 2.00]**	1.31 [0.84, 2.04]	1.24 [0.77, 2.00]	**1.55** * **[1.01, 2.40]**	1.53 [0.98, 2.38]	**5.08** *** **[2.52, 10.24]**	**4.94** *** **[2.44, 9.99]**	1.03 [0.72, 1.47]	1.02 [0.71, 1.46]
Q5 (very high)	**1.43** * **[1.05, 1.94]**	**1.39** * **[1.01, 1.91]**	1.41 [0.91, 2.17]	1.45 [0.91, 2.32]	1.36 [0.87, 2.11]	1.31 [0.83, 2.04]	**2.89** ** **[1.49, 5.60]**	**2.86** ** **[1.47, 5.56]**	1.13 [0.79, 1.61]	1.10 [0.77, 1.58]
*p* trend	**0.016**	**0.024**	0.193	0.149	**0.025**	**0.036**	**<0.001**	**<0.001**	0.683	0.764

*Note*: Neighborhood gentrification represents the sum of *z* scores of percent change of three socioeconomic indicators (i.e., college or more educated adults aged 25 or more, number of residents living below the federal poverty line, and median household income). The index was operationalized as continuous (higher scores indicates greater gentrification) and quintiles. Model 1 is adjusted for age at study entry (continuous), aspirin use (yes/no), family history of prostate cancer (first‐degree relatives, yes/no), diabetes history (yes/no), body mass index at study entry (continuous), self‐reported race (African American, European American, not included in race stratified analyses), smoking status (current, former, never, and missing). Model 2 additionally adjusted for education (high school or less, some college, college, professional school, and missing), individual income (not included in the income stratified analyses, <$30,000, $30,000–$60,000, $60,000–$90,000, >$90,000). The 95% confidence interval defines significance.

Abbreviations: AA, African American; CI, confidence interval; EA, European American; OR, odds ratio.

* *p* < 0.05

** *p* < 0.01

*** *p* < 0.001.

### 3.3 Association of neighborhood gentrification with National Comprehensive Cancer Network (NCCN)‐defined risk scores

**FIGURE 2:**
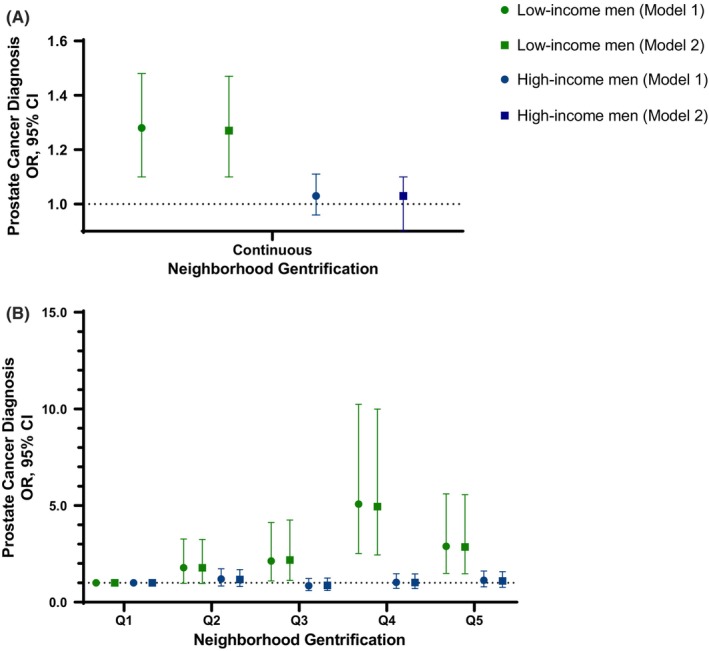
Association of neighborhood gentrification with a prostate cancer diagnosis stratified by income. Odds ratios (OR) and 95% confidence interval (CI) for a prostate cancer diagnosis by neighborhood gentrification with gentrification coded as (A) continuous score and (B) quintiles, with additional stratification by self‐reported income. Low income = <$30,000, high income = ≥$30,000. Logistic regression with Model 1 being adjusted for all baseline covariates, whereas Model 2 adjusted for all covariates with the additional inclusion of education.

Next, we asked the question if gentrification (continuous) is associated with NCCN‐defined risk groups for prostate cancer patients. There was no association linking increased gentrification to NCCN risk scores among all men and in race/stratified analysis (see Table S3). This pattern was principally replicated in a follow‐up sensitivity analysis, where NCCN risk categories were dichotomized into NCCN‐defined localized (low‐, intermediate‐, high‐, and very high‐risk score) versus regional/distant metastatic disease (Table S4). Among EA men, increased gentrification was associated with decreased odds of presenting with NCCN‐defined regional/metastatic prostate cancer in the SES‐adjusted analysis (OR model 2: 0.61, 95% CI: 0.40–0.94) (Table S4; Figure 2). No association was found in the income‐stratified analysis.

### 3.4 Relationships between neighborhood gentrification and serum proteomics‐defined biological pathways related to inflammation and immune function

We previously reported an association of neighborhood deprivation with 82 circulating immune‐oncology markers and several biological pathways defined by them.^7^ To evaluate whether the biological pathways are also influenced by gentrification, we examined potential associations of gentrification with three pathways, namely inflammation, chemotaxis, and suppression of tumor immunity, as those were the pathways whose activity scores were found to be associated with neighborhood deprivation.^7^ We restricted this analysis to the control population, allowing us to exclude a confounding effect of prostate cancer. Using MANOVA regression, we did not find that gentrification coded as either continuous variable or stratified into quintiles was associated with the activity scores for the three pathways (Tables S5 and S6; Figures S1–S3). Hence, in contrast to cross‐sectional neighborhood deprivation, gentrification seem not to have an influence on these serum proteome‐defined pathways.

### 3.5 Neighborhood gentrification and mortality after a prostate cancer diagnosis

We investigated whether gentrification may predict the occurrence of prostate cancer mortality, using an analysis of all‐cause and prostate cancer‐specific mortality. The analysis, applying multivariable Cox regression modeling with gentrification coded as a continuous variable or stratified into quintiles, did not reveal consistent associations or trends for the relationship between gentrification and a prostate cancer mortality (Table S7; Figure S4). Gentrification was not associated with all‐cause or prostate cancer‐specific mortality after a prostate cancer diagnosis, among all patients or in the race/ethnicity‐stratified analysis. Still, follow‐up sensitivity analysis (Table 2) indicated that gentrification may be associated with all‐cause mortality (HR model 1: 1.11, 95% CI: 1.03–1.21; HR model 2: 1.10, 95% CI: 1.01–1.20) and prostate cancer‐specific mortality (SHR model 1: 1.15, 95% CI: 1.01–1.31; SHR model 2: 1.16, 95% CI: 1.01–1.33) of prostate cancer patients who presented with localized disease at time of disease diagnosis among all men. In non‐SES‐adjusted models stratified by race/ethnicity, AA men, who presented with localized disease at time of disease diagnosis, experienced higher risk of an all‐cause (HR model 1: 1.11, 95% CI: 1.01–1.22) and prostate cancer‐specific mortality (SHR model 1: 1.18, 95% CI: 1.01–1.38) with increased gentrification. We did not observe the same association with EA men. Lastly, low‐income men also experienced a higher risk of an all‐cause mortality (HR model 2: 1.16, 95% CI: 1.02–1.33) and prostate cancer‐specific mortality (CSHR model 2: 1.54, 95% CI: 1.11–2.14; SHR model 2: 1.45, 95% CI: 1.11–1.90), with or without further adjustment for education.

**TABLE 2:** Association between neighborhood gentrification and all‐cause and disease‐specific mortality among African and European American men with prostate cancer, by disease stage.

	AA + EA men, *n* = 769		AA men, *n* = 380		EA men, *n* = 349		Low‐income men, *n* = 264		High‐income men, *n* = 465	
Median survival time, years	5.92		5.62		6.24		5.07		6.77	
	Model 1	Model 2	Model 1	Model 2	Model 1	Model 2	Model 1	Model 2	Model 1	Model 2
Mortality outcomes										
All‐cause mortality, HR [95% CI]										
Localized disease, *n* = 729	**1.11** * **[1.03, 1.21]**	**1.10** * **[1.01, 1.20]**	**1.11** * **[1.01, 1.22]**	1.09 [0.99, 1.20]	1.10 [0.95, 1.28]	1.12 [0.96, 1.29]	**1.15** * **[1.01, 1.32]**	**1.16** * **[1.02, 1.33]**	1.08 [0.96, 1.22]	1.08 [0.95, 1.21]
Regional or metastatic disease, *n* = 40	1.05 [0.70, 1.58]	1.28 [0.65, 2.51]	‐	‐	‐	‐	‐	‐	‐	‐
Prostate cancer‐specific mortality, Cox Regression, CSHR [95% CI]										
Localized disease, *n* = 729	1.18 [0.99, 1.41]	1.18 [0.99, 1.42]	1.20 [0.97, 1.48]	1.18 [0.95, 1.47]	1.14 [0.79, 1.64]	1.22 [0.87, 1.72]	**1.42** * **[1.04, 1.94]**	**1.54** ** **[1.11, 2.14]**	1.07 [0.77, 1.48]	1.06 [0.77, 1.48]
Regional or metastatic disease, *n* = 40	1.28 [0.80, 2.05]	1.68 [0.77, 3.66]	‐	‐	‐	‐	‐	‐	‐	‐
Prostate cancer‐specific mortality, Fine and Gray Regression, SHR [95% CI]										
Localized disease, *n* = 729	**1.15** * **[1.01, 1.31]**	**1.16** * **[1.01, 1.33]**	**1.18** * **[1.01, 1.38]**	1.16 [0.94, 1.43]	1.12 [0.83, 1.50]	1.20 [0.94, 1.52]	**1.33** * **[1.07, 1.66]**	**1.45** ** **[1.11, 1.90]**	1.07 [0.84, 1.36]	1.08 [0.85, 1.39]
Regional or metastatic disease, *n* = 40	1.13 [0.74, 1.74]	1.16 [0.51, 2.63]	‐	‐	‐	‐	‐	‐	‐	‐

*Note*: Neighborhood gentrification represents the sum of *z* scores of percent change of three socioeconomic indicators (i.e., college or more educated adults aged 25 or more, number of residents living below the federal poverty line, and median household income). The index was operationalized as continuous (where higher scores indication greater gentrification). Disease stage was defined according to the National Comprehensive Cancer Network risk score categories localized (low, intermediate, high/very high categories) versus regional or metastatic. Model 1, Cox regression analysis adjusted for age at study entry (continuous), aspirin use (yes/no), family history of prostate cancer (first‐degree relatives, yes/no), diabetes history (yes/no), body mass index at study entry (continuous), self‐reported race (not included in race stratified analyses, African American, and European American), smoking status (current, former, never, and missing), treatment (none, surgery, radiation, hormone, combination, and missing). Model 2 additionally adjusted for education (high school or less, some college, college, professional school, and missing), individual income (not included in income stratified analyses <$30,000, $30,000–$60,000, $60,000–$90,000, >$90,000, missing). Low‐income men defined as <$30,000 and middle‐/high‐income men as ≥$30,000. The race and income stratified analysis was only conducted among those presenting with localized disease. The 95% confidence interval defines significance.

Abbreviations: AA, African American; CI, confidence interval; CSHR, cause‐specific hazard ratio; EA, European American; HR, hazard ratio; SHR, subdistribution hazard ratio.

* *p* < 0.05

** *p* < 0.01.

## 4 DISCUSSION

In this diverse case–control study, we investigated the relationship of neighborhood gentrification (i.e., change in socioeconomic characteristics—education, poverty, and income) with a prostate cancer diagnosis and all‐cause and disease‐specific mortality. We found that increasing gentrification was associated with prostate cancer in our study.

Among long‐standing residents and historically underserved racial/ethnic groups, gentrification limits access to adequate health care, resources to purchase healthy foods, and affordable housing.^11^, ^17^, ^26^ Incoming residents within gentrifying neighborhoods are more likely to be wealthier, White, have a higher level of formal education, and able to afford new or renovated homes^17^, ^27^ compared to long standing residents. Studies suggest that gentrification disrupts access to adequate primary healthcare and increases competition for scarce resources among long‐standing and displaced community members,^10^, ^18^, ^28^ particularly preventive medical services tailored for racial/ethnic underserved communities.^12^ Second, although gentrification increases the number and quality of parks, recreational areas, and supermarkets,^17^, ^29^, ^30^ affluent residents are more likely to reap these benefits.^10^, ^26^, ^31^ Third, gentrification is posited as a potential carcinogenic attribute, due to increases in cancer risk factors within gentrifying neighborhoods compared to disadvantaged neighborhoods that did not gentrify, particularly resulting from increases in vehicle density with the inflow of White residents.^32^ Lastly, gentrification destabilizes long standing communities, fosters physical and sociocultural (e.g., social network and community cohesion) displacement as well as resegregation, and decreases neighborhood‐related feelings of safety and residential stability due to rising rent costs, all of which over the long term produce chronic stress.^33^, ^34^ Overall, perpetuation of structural inequities resulting from gentrification may limit engagement in cancer preventive behaviors and lead to related stress‐induced pro‐inflammatory signaling and alterations of gene expression, fostering immune microenvironments that promote tumor development, among long‐standing racial/ethnic residents of gentrifying communities.^9^

The association of gentrification with human health and disease has been studied for a broad array of health endpoints.^10^, ^17^ However, none of these studies investigated the relationship with prostate cancer.^10^, ^17^ Thus, our study is the first to examine the influence of gentrification on prostate cancer risk and mortality, and prostate cancer‐related biological pathways, using a diverse case–control study conducted in Baltimore, Maryland, and surrounding areas. Our observations revealed that, among all men combined, high levels of gentrification were associated with a prostate cancer diagnosis in the SES‐adjusted analysis. AA men, however, experienced the highest odds of prostate cancer when residing within tracts with moderate levels of gentrification. We also found that low‐income men, but not high‐income men, experienced an increased risk of prostate cancer with increased gentrification, with the highest odds when residing in tracts with moderate gentrification. The latter findings allude to an important role of individual SES within gentrifying neighborhoods in defining prostate cancer risk.

Multiple systematic reviews revealed mixed health effects of gentrification/socioeconomic ascent.^10^, ^17^, ^35^ One previous study found no association of gentrification with colorectal cancer risk.^36^ Yet, in line with our current study of prostate cancer, systematic reviews showed that the health effects of gentrification were particularly impactful for AA and low‐income residents when compared with EA residents and higher income residents.^10^, ^17^, ^37^ As mentioned above stressors and structural inequities resulting from gentrification may increase risk of prostate cancer development among AA and low‐income men; however, it is also plausible that increased diagnosis of prostate cancer among of residents of gentrifying neighborhoods may reflect higher access to prostate cancer screening and early detection.

Of note, AA with lower rates of education were diagnosed with prostate cancer at higher rates compared to those with higher education, perhaps due to effects of past discrimination and injustice that correlate with low educational attainment and disease risk. These findings align with a systematic review that found that prostate cancer primarily affects AA men, and those reporting lower socioeconomic status and educational attainment.^38^ However, in our cohort this pattern was not observed among EA. One‐third of EA cases reported high levels of income, which translates to greater access to screenings.^39^ These patterns are important to consider within the context of gentrification as they highlight the exponential impact of low SES in increasing prostate cancer risk among AA, which may compound with gentrification‐related processes. However, in our study we were underpowered to examine interactions between race and income within the context of gentrification.

Our study also examined how gentrification may relate to NCCN‐defined risk groups and prostate cancer mortality among AA and EA men. We found that EA but not AA men may experience significantly reduced odds of regional or metastatic cancer when residing in neighborhoods with increasing gentrification. This observation seem to counter a prior report that found that census tract‐defined upward socioeconomic mobility was associated with metastatic breast cancer.^40^ Additionally, previous studies found an association between upward neighborhood socioeconomic mobility and decreased cancer mortality.^41^ In our cohort, gentrification was associated with all‐cause and prostate cancer‐specific mortality among AA and low‐income men that presented with localized prostate cancer at the time of diagnosis. Among EA, gentrification was not associated with lethal prostate cancer. It is important to note that we did not further stratify our analysis by residential tenure that are indicators for cancer outcomes within the context of gentrification.

Among EA, upward neighborhood mobility in redlined/racially segregated communities may translate to increased access to medical resources aimed at detecting prostate cancer in its early stages; however, as previously discussed, this developing resource advantage may not extend to AA men. Gentrifying neighborhoods are theorized to undergo an increase in health promoting amenities and services, particularly targeted toward incoming residents who are often of middle/high socioeconomic standing and/or White.^12^, ^42^, ^43^ Long‐standing residents of gentrifying areas are usually lower income, racial/ethnic minorities, and/or uninsured or publicly insured, and are often unable to access new medical resources that cater to wealthier and privately insured patients.^12^, ^44^ At the same time gentrifying neighborhoods experience closings or replacement of safety‐net hospitals that primarily served the AA community, publicly insured, and uninsured patient populations.^12^ Among AA, gentrification may exacerbate inequities in preventive services access (e.g., Prostate‐Specific Antigen tests), and increase risk for cancer progression and mortality among patients with localized prostate cancer and those with mild prostate cancer‐related symptomology.^45^, ^46^ Lastly, medical mistrust, medical discrimination, adverse physician–patient communication, and health insurance access, and stigma, rooted in historical racism among AA communities, may play a large role in the under‐use of medical resources introduced by gentrification.^47^, ^48^, ^49^

We previously reported that neighborhood deprivation may increase peripheral inflammation, chemotaxis, and suppression of tumor immunity.^7^ Therefore, we examined if gentrification may also associate with the activity of these three‐serum proteome‐defined pathways but did not find robust associations. As suggested by prior work, gentrification‐related neighborhood improvements may have small effects in the context of cancer.^36^ A longer latency period^36^ or the examinations residential displacement from gentrifying neighborhoods, among long‐term residents, may be needed to observe effects related to serum proteome‐defined pathways and mortality. It is plausible that prior cumulative exposure to neighborhood deprivation may muddle the effects of improvements related to gentrification or be a stronger predictor of peripheral inflammation, chemotaxis, and suppression of tumor immunity, and all‐cause and prostate‐related mortality. Lastly, other oncology‐related markers or neighborhood dimensions of neighborhood environments may be of more relevance.

Our study provided first evidence for a link between gentrification and prostate cancer and has strength. It is a large and diverse case control study with long term follow‐up for all‐cause and disease‐specific deaths and measurement of immune‐oncology markers. Geocoded addresses at baseline allowed us to construct a previously used measure of neighborhood gentrification. Despite the unique strengths, there are also limitations. First, collection of cases, controls, immune‐oncology proteins, and associated pathways occurred from 2005 to 2015, while the gentrification index was created for the years 1990 to 2006–2010, which may lead to uncertainty in results due to differences in time frame of exposure and outcome. Additionally, we only collected residential addresses at the time of recruitment, thus we were not able to capture the effects of the duration of exposure across the life course, account for residential tenure or examine longitudinal associations due to the cross‐sectional nature of the study. The lack of historical addresses and residential mobility after the baseline visit may have led to misclassification of neighborhood gentrification over time. We were limited in capturing the full gentrification process—when neighborhoods started to experience any gentrification‐related process, whether gentrification has continued or reached a stable state, and the magnitude of these processes. Also, we were unable to capture the effects of displacement, an important mechanism in the association between gentrification and health.^17^ As the gentrification literature is relatively new and growing, standardized approaches to measuring and capturing multiple dimensions of neighborhood change over time will be crucial to truly understand the gentrification process and its role on biological processes and health outcomes. The present study provides initial insights to the potential role of gentrification on cancer and hopes to stimulate further inquiries in this area.

## 5 CONCLUSION

Our study suggests that neighborhood gentrification is associated with prostate cancer risk and prostate cancer survival in a diverse population of men from the greater Baltimore area in Maryland, albeit associations were heterogenous within subgroups. The role of gentrification on serum proteome‐defined chemotaxis, inflammation, and tumor immunity suppression remains unclear, based on our findings. The data suggest that changing neighborhood socioeconomic environments may be linked to cancer risks and outcomes through complex mechanisms, that vary for AA and EA men.

## AUTHOR CONTRIBUTIONS

**Catherine Pichardo:** Conceptualization (equal); data curation (equal); formal analysis (lead); investigation (equal); methodology (equal); validation (lead); visualization (lead); writing – original draft (lead); writing – review and editing (lead). **Adaora Ezeani:** Conceptualization (supporting); formal analysis (equal); investigation (supporting); methodology (supporting); validation (equal); writing – original draft (supporting); writing – review and editing (supporting). **Margaret S. Pichardo:** Conceptualization (supporting); data curation (supporting); formal analysis (supporting); investigation (supporting); methodology (supporting); writing – review and editing (supporting). **Tanya Agurs‐Collins:** Conceptualization (supporting); formal analysis (supporting); supervision (equal); writing – review and editing (supporting). **Tiffany M. Powell‐Wiley:** Conceptualization (supporting); writing – review and editing (supporting). **Brid Ryan:** Conceptualization (supporting); writing – review and editing (supporting). **Tsion Zewdu Minas:** Data curation (supporting); formal analysis (supporting); investigation (supporting); methodology (supporting); validation (supporting); writing – review and editing (supporting). **Maeve Bailey‐Whyte:** Conceptualization (supporting); formal analysis (supporting); investigation (supporting); methodology (supporting); validation (supporting); writing – review and editing (supporting). **Wei Tang:** Data curation (supporting); investigation (supporting); methodology (supporting); validation (supporting); writing – review and editing (supporting). **Tiffany H. Dorsey:** Data curation (equal); investigation (supporting); methodology (supporting); validation (supporting); writing – review and editing (supporting). **William Wooten:** Data curation (supporting); writing – review and editing (supporting). **Christopher A. Loffredo:** Formal analysis (supporting); investigation (supporting); methodology (supporting); writing – review and editing (supporting). **Stefan Ambs:** Conceptualization (equal); data curation (supporting); formal analysis (supporting); funding acquisition (lead); investigation (lead); methodology (equal); project administration (lead); resources (lead); software (supporting); supervision (lead); validation (equal); visualization (supporting); writing – original draft (supporting); writing – review and editing (equal).

## FUNDING INFORMATION

This work was supported by the Intramural Research Program of the NIH, National Cancer Institute (NCI), Center for Cancer Research (ZIA BC 010499, 010624 to S.A.), and DoD award W81XWH1810588 (to S.A.). Dr. Powell‐Wiley is supported by the Division of Intramural Research at the National Heart, Lung, and Blood Institute (ZIA HL006168; ZIA HL006225; ZIA HL006252) and the Intramural Research Program of the NIMHD (ZIJ MD000010).

## CONFLICT OF INTEREST STATEMENT

The authors declare no potential conflicts of interest.

## ETHICS STATEMENT

The National Cancer Institute's (protocol# 05‐C‐N021) and the University of Maryland's (protocol #0298229) Institutional Review Boards approved the study, and both recruitment and research followed the ethical guidelines set by the Declaration of Helsinki. All patients provided written informed consent.

## Supporting information


Data S1:

## Data Availability

Clinical, demographic, and molecular data (serum proteome data) for the NCI‐Maryland prostate cancer study have been deposited at the Open Science Framework at https://doi.org/10.17605/OSF.IO/327HA and as a public GitHub Repository at https://doi.org/10.5281/zenodo.5815262. The remaining data are available from the authors upon request. Personal identifiers such as neighborhood census tract data cannot be shared.

## References

[1] Tsodikov A , Gulati R , de Carvalho TM , et al. Is prostate cancer different in black men? Answers from 3 natural history models. Cancer. 2017;123(12):2312‐2319. doi:10.1002/cncr.30687 28436011 PMC5459620

[2] Seewaldt VL , Winn RA . Residential racial and economic segregation and cancer mortality in the US—speaking out on inequality and injustice. JAMA Oncol. 2023;9(1):126‐127. doi:10.1001/jamaoncol.2022.5272 36394869

[3] Landrine H , Corral I , Lee JGL , Efird JT , Hall MB , Bess JJ . Residential segregation and racial cancer disparities: a systematic review. J Racial Ethn Health Disparities. 2017;4(6):1195‐1205. doi:10.1007/s40615-016-0326-9 28039602

[4] Diez Roux AV , Mair C . Neighborhoods and health. Ann N Y Acad Sci. 2010;1186(1):125‐145. doi:10.1111/j.1749-6632.2009.05333.x 20201871

[5] Landrine H , Corral I . Separate and unequal: residential segregation and black health disparities. Ethn Dis. 2009;19(2):179‐184. 19537230

[6] Sorice KA , Fang CY , Wiese D , et al. Systematic review of neighborhood socioeconomic indices studied across the cancer control continuum. Cancer Med. 2022;11(10):2125‐2144. doi:10.1002/cam4.4601 35166051 PMC9119356

[7] Pichardo MS , Minas TZ , Pichardo CM , et al. Association of neighborhood deprivation with prostate cancer and immune markers in African American and European American men. JAMA Netw Open. 2023;6(1):e2251745. doi:10.1001/jamanetworkopen.2022.51745 36662526 PMC9860532

[8] Gomez SL , Shariff‐Marco S , DeRouen M , et al. The impact of neighborhood social and built environment factors across the cancer continuum: current research, methodological considerations, and future directions. Cancer. 2015;121(14):2314‐2330. doi:10.1002/cncr.29345 25847484 PMC4490083

[9] Rebbeck TR . Conquering cancer disparities: new opportunities for cancer epidemiology, biomarker, and prevention research. Cancer Epidemiol Biomarkers Prev. 2006;15(9):1569‐1571. doi:10.1158/1055-9965.Epi-06-0613 16985011

[10] Bhavsar NA , Kumar M , Richman L . Defining gentrification for epidemiologic research: a systematic review. PLoS One. 2020;15(5):e0233361. doi:10.1371/journal.pone.0233361 32437388 PMC7241805

[11] Cole HVS , Mehdipanah R , Gullón P , Triguero‐Mas M . Breaking down and building up: gentrification, its drivers, and urban health inequality. Current Environ Health Rep. 2021;8(2):157‐166. doi:10.1007/s40572-021-00309-5 PMC7955692 33713334

[12] Cole HVS , Franzosa E . Advancing urban health equity in the United States in an age of health care gentrification: a framework and research agenda. Int J Equity Health. 2022;21(1):66. doi:10.1186/s12939-022-01669-6 35546673 PMC9092322

[13] Ding L , Hwang J , Divringi E . Gentrification and residential mobility in Philadelphia. Reg Sci Urban Econ. 2016;61:38‐51. doi:10.1016/j.regsciurbeco.2016.09.004 28579662 PMC5450830

[14] Tulier ME , Reid C , Mujahid MS , Allen AM . "Clear action requires clear thinking": a systematic review of gentrification and health research in the United States. Health Place. 2019;59:102173. doi:10.1016/j.healthplace.2019.102173 31357049 PMC6868313

[15] Kennedy M , Leonard P . Gentrification: practice and politics. Home Ownership Summit 2000 Research Series. Washington, DC. 2001.

[16] Li BY . Now is the time: challenging resegregation and displacement in the age of hypergentrification. Fordham Law Rev. 2016;85:1189.

[17] Schnake‐Mahl AS , Jahn JL , Subramanian SV , Waters MC , Arcaya M . Gentrification, neighborhood change, and population health: a systematic review. J Urban Health. 2020;97(1):1‐25. doi:10.1007/s11524-019-00400-1 31938975 PMC7010901

[18] Betancur JJ . The politics of gentrification: the case of west town in Chicago. Urban Aff Rev. 2002;37(6):780‐814. doi:10.1177/107874037006002

[19] Smith CJ , Dorsey TH , Tang W , Jordan SV , Loffredo CA , Ambs S . Aspirin use reduces the risk of aggressive prostate cancer and disease recurrence in African‐American men. Cancer Epidemiol Biomarkers Prev. 2017;26(6):845‐853. doi:10.1158/1055-9965.EPI-16-1027 28292923 PMC5457351

[20] Kiely M , Milne GL , Minas TZ , et al. Urinary thromboxane B2 and lethal prostate cancer in African American men. J Natl Cancer Inst. 2021;114:123‐129. doi:10.1093/jnci/djab129 PMC8755482 34264335

[21] Geolytics . Neighborhood change database [NCDB] tract data from 1970–2010 [Online demographic data]. 2014.

[22] Freeman L , Braconi F . Gentrification and displacement New York City in the 1990s. J Am Plann Assoc. 2004;70(1):39‐52. doi:10.1080/01944360408976337

[23] Huynh M , Maroko AR . Gentrification and preterm birth in New York City, 2008–2010. J Urban Health. 2014;91(1):211‐220. doi:10.1007/s11524-013-9823-x 24022181 PMC3907632

[24] Linton SL , Cooper HLF , Kelley ME , et al. Cross‐sectional association between ZIP code‐level gentrification and homelessness among a large community‐based sample of people who inject drugs in 19 US cities. BMJ Open. 2017;7(6):e013823. doi:10.1136/bmjopen-2016-013823 PMC5541298 28637724

[25] Minas TZ , Candia J , Dorsey TH , et al. Serum proteomics links suppression of tumor immunity to ancestry and lethal prostate cancer. Nat Commun. 2022;13(1):1759. doi:10.1038/s41467-022-29235-2 35365620 PMC8975871

[26] Anguelovski I . Healthy food stores, greenlining and food gentrification: contesting new forms of privilege, displacement and locally unwanted land uses in racially mixed neighborhoods. Int J Urban Reg Res. 2015;39(6):1209‐1230. doi:10.1111/1468-2427.12299

[27] Rucks‐Ahidiana Z . Racial composition and trajectories of gentrification in the United States. Urban Studies. 2021;58(13):2721‐2741. doi:10.1177/0042098020963853

[28] Development USDoHaU , Research OoPDa . Displacement of lower‐income families in urban areas report. 2018.

[29] Byrne JP . Two cheers for gentrification. Howard Law J. 2002;46(3):405‐432.

[30] Mullenbach LE , Baker BL . Environmental justice, gentrification, and leisure: a systematic review and opportunities for the future. Leis Sci. 2020;42(5–6):430‐447. doi:10.1080/01490400.2018.1458261

[31] Cole HVS , Triguero‐Mas M , Connolly JJT , Anguelovski I . Determining the health benefits of green space: does gentrification matter? Health Place. 2019;57:1‐11. doi:10.1016/j.healthplace.2019.02.001 30844594

[32] Schachner JN . Is gentrification a carcinogen? Neighborhood change and cancerous vehicle emissions in Los Angeles County. Hous Policy Debate. 2022;33:1‐25. doi:10.1080/10511482.2022.2099936

[33] Betancur J . Gentrification and community fabric in Chicago. Urban Stud. 2011;48(2):383‐406. doi:10.1177/0042098009360680 21275200

[34] Tran LD , Rice TH , Ong PM , Banerjee S , Liou J , Ponce NA . Impact of gentrification on adult mental health. Health Serv Res. 2020;55(3):432‐444. doi:10.1111/1475-6773.13264 31957022 PMC7240775

[35] Smith GS , Breakstone H , Dean LT , Thorpe RJ Jr . Impacts of gentrification on health in the US: a systematic review of the literature. J Urban Health. 2020;97(6):845‐856. doi:10.1007/s11524-020-00448-4 32829469 PMC7704880

[36] Zhang D , Matthews CE , Powell‐Wiley TM , Xiao Q . Ten‐year change in neighborhood socioeconomic status and colorectal cancer. Cancer. 2019;125(4):610‐617. doi:10.1002/cncr.31832 30423200 PMC7135907

[37] Smith GS , Thorpe RJ Jr . Gentrification: a priority for environmental justice and health equity research. Ethn Dis. 2020;30(3):509‐512. doi:10.18865/ed.30.3.509 32742156 PMC7360181

[38] Patki S , Aquilina J , Thorne R , et al. A systematic review of patient race, ethnicity, socioeconomic status, and educational attainment in prostate cancer treatment randomised trials—is the evidence base applicable to the general patient population? Eur Urol Open Sci. 2023;54:56‐64. doi:10.1016/j.euros.2023.05.015 37545851 PMC10403690

[39] Rundle A , Neckerman KM , Sheehan D , et al. A prospective study of socioeconomic status, prostate cancer screening and incidence among men at high risk for prostate cancer. Cancer Causes Control. 2013;24(2):297‐303. doi:10.1007/s10552-012-0108-6 23224323 PMC3557724

[40] Barrett RE , Cho YI , Weaver KE , et al. Neighborhood change and distant metastasis at diagnosis of breast cancer. Ann Epidemiol. 2008;18(1):43‐47. doi:10.1016/j.annepidem.2007.07.001 17890103

[41] Xiao Q , Berrigan D , Powell‐Wiley TM , Matthews CE . Ten‐year change in neighborhood socioeconomic deprivation and rates of total, cardiovascular disease, and cancer mortality in older US adults. Am J Epidemiol. 2018;187(12):2642‐2650. doi:10.1093/aje/kwy181 30137194 PMC6269245

[42] Yin D , Morris C , Allen M , Cress R , Bates J , Liu L . Does socioeconomic disparity in cancer incidence vary across racial/ethnic groups? Cancer Causes Control. 2010;21(10):1721‐1730. doi:10.1007/s10552-010-9601-y 20567897 PMC2941051

[43] Poulson MR , Kenzik KM , Singh S , et al. Redlining, structural racism, and lung cancer screening disparities. J Thorac Cardiovasc Surg. 2022;163(6):1920‐1930.e2. doi:10.1016/j.jtcvs.2021.08.086 34774325

[44] Lim S , Chan PY , Walters S , Culp G , Huynh M , Gould LH . Impact of residential displacement on healthcare access and mental health among original residents of gentrifying neighborhoods in New York City. PLoS One. 2017;12(12):e0190139. doi:10.1371/journal.pone.0190139 29272306 PMC5741227

[45] Moses KA , Orom H , Brasel A , Gaddy J , Underwood W III . Racial/ethnic disparity in treatment for prostate cancer: does cancer severity matter? Urology. 2017;99:76‐83. doi:10.1016/j.urology.2016.07.045 27667157 PMC5191943

[46] Sundi D , Faisal FA , Trock BJ , et al. Reclassification rates are higher among African American men than Caucasians on active surveillance. Urology. 2015;85(1):155‐160. doi:10.1016/j.urology.2014.08.014 25440814 PMC4275346

[47] Vapiwala N , Miller D , Laventure B , et al. Stigma, beliefs and perceptions regarding prostate cancer among Black and Latino men and women. BMC Public Health. 2021;21(1):758. doi:10.1186/s12889-021-10793-x 33879107 PMC8056613

[48] Lillard JW Jr , Moses KA , Mahal BA , George DJ . Racial disparities in Black men with prostate cancer: a literature review. Cancer. 2022;128(21):3787‐3795. doi:10.1002/cncr.34433 36066378 PMC9826514

[49] Kinlock BL , Thorpe RJ Jr , Howard DL , et al. Racial disparity in time between first diagnosis and initial treatment of prostate cancer. Cancer Control. 2016;23(1):47‐51. doi:10.1177/107327481602300108 27009456 PMC6448564

