# Supplementary material for: Association of neighborhood gentrification with prostate cancer and immune markers in African American and European American men
Source: Cancer Med. 2023 Dec 27;13(1):e6828. doi: 10.1002/cam4.6828 (PMC10807554; doi:10.1002/cam4.6828)
Supplement: Supplementary file 1 — Data S1: [file CAM4-13-e6828-s001.docx]

**eMethods.**

*Covariates.* Individual level socio-demographic indicators and health information were collected utilizing surveys and medical records. Our analyses were controlled for age at study entry (continuous), aspirin use (yes/no), family history of prostate cancer (first-degree relatives, yes/no), diabetes history (yes/no), body mass index at study entry (continuous), self-reported race (African American, European American), West African Ancestry markers (in systemic inflammation and immune function only), smoking status (current, former, never), education (high school or less, some college, college, professional school), and annual income (< $10,000, $10,000–$30,000, $30,000–$60,000, $60,000–$90,000, >$90,000). Models of survival additionally controlled for treatment (none, surgery, radiation, hormone) and the National Comprehensive Cancer Network (NCCN) risk scores. We used two models in our analysis. In model 1, we adjusted for all covariates without SES, whereas in model 2 we added SES (i.e., income and education) to the model 1 covariates. Missing values for education (n = 2) and smoking (n = 16) were categorized as the highest level for each variable.

**Supplementary Table 1. Descriptive characteristics of African American men in the NCI-Maryland Case-Control Study, by neighborhood gentrification quintiles**

|  | **African American cases, n = 405** | | | | | | | **African American controls, n = 479** | | | | | | |
| --- | --- | --- | --- | --- | --- | --- | --- | --- | --- | --- | --- | --- | --- | --- |
|  | Total | Very low | Low | Moderate | High | Very High | *P* value | Total | Very low | Low | Moderate | High | Very High | *P* value |
| **Individual-level Characteristics** |  | n = 81 | n = 115 | n = 76 | n = 62 | n = 71 |  |  | n = 133 | n = 107 | n = 72 | n = 80 | n = 87 |  |
| Age, years, mean (SD) | 62.61 (7.32) | 61.95 (7.23) | 62.36 (7.21) | 62.21 (7.19) | 63.44 (7.48) | 63.49 (7.68) | 0.587 | 64.53 (7.79) | 63.88 (7.75) | 64.71 (7.54) | 63.29 (7.01) | 64.19 (7.90) | 66.67 (8.42) | **0.049** |
| Education, n (%) |  |  |  |  |  |  | 0.912 |  |  |  |  |  |  | **0.034** |
| High school or less | 192 (47.41) | 39 (48.15) | 56 (48.70) | 33 (43.42) | 31 (50) | 33 (46.48) |  | 143 (29.85) | 29 (21.8) | 37 (34.58) | 21 (29.17) | 27 (33.75) | 29 (33.33) |  |
| Some college | 150 (37.04) | 30 (37.04) | 44 (38.26) | 26 (34.21) | 23 (37.10) | 27 (38.03) |  | 142 (29.65) | 45 (33.83) | 37 (34.58) | 24 (33.33) | 17 (21.25) | 19 (21.84) |  |
| College | 44 (10.86) | 9 (11.11) | 11 (9.57) | 12 (15.79) | 6 (9.68) | 6 (8.45) |  | 105 (21.92) | 40 (30.08) | 17 (15.89) | 10 (13.89) | 19 (23.75) | 19 (21.84) |  |
| Professional school | 18 (4.44) | 3 (3.70) | 4 (3.48) | 4 (5.26) | 2 (3.23) | 5 (7.04) |  | 88 (18.37) | 19 (14.29) | 16 (14.95) | 16 (22.22) | 17 (21.25) | 20 (22.99) |  |
| Missing | 1 (0.25) | 0 (0) | 0 (0) | 1 (1.32) | 0 (0) | 0 (0) |  | 1 (0.21) | 0 (0) | 0 (0) | 1 (1.39) | 0 (0) | 0 (0) |  |
| Income, n (%) |  |  |  |  |  |  | **0.008** |  |  |  |  |  |  | 0.059 |
| < $10,000 | 205 (50.62) | 33 (40.74) | 54 (46.96) | 37 (48.68) | 40 (64.52) | 41 (57.75) |  | 112 (23.38) | 33 (24.81) | 36 (33.64) | 14 (19.44) | 14 (17.5) | 15 (17.24) |  |
| $10,000-$30,000 | 92 (22.72) | 14 (17.28) | 37 (32.17) | 18 (23.68) | 12 (19.35) | 11 (15.49) |  | 105 (21.92) | 27 (20.30) | 24 (22.43) | 14 (19.44) | 16 (20.00) | 24 (27.59) |  |
| $30,000-$60,000 | 40 (9.88) | 15 (18.52) | 9 (7.83) | 5 (6.58) | 4 (6.45) | 7 (9.86) |  | 109 (22.76) | 37 (27.82) | 24 (22.43) | 16 (22.22) | 15 (18.75) | 17 (19.54) |  |
| $60,000-$90,000 | 29 (7.16) | 8 (9.88) | 3 (2.61) | 6 (7.89) | 4 (6.45) | 8 (11.27) |  | 107 (22.34) | 26 (19.55) | 12 (11.21) | 21 (29.17) | 24 (30.00) | 24 (27.59) |  |
| > $90,000 | 39 (9.63) | 11 (13.58) | 12 (10.43) | 10 (13.16) | 2 (3.23) | 4 (5.63) |  | 46 (9.6) | 10 (7.52) | 11 (10.28) | 7 (9.72) | 11 (13.75) | 7 (8.05) |  |
| Aspirin use, n (%) | 170 (41.98) | 30 (37.04) | 50 (43.48) | 32 (42.11) | 25 (40.32) | 33 (46.48) | 0.813 | 247 (51.57) | 62 (46.62) | 60 (56.07) | 44 (61.11) | 36 (45.00) | 45 (51.72) | 0.186 |
| Has family history of prostate cancer, n (%) | 43 (10.62) | 11 (13.58) | 13 (11.30) | 5 (6.58) | 8 (12.90) | 6 (8.45) | 0.591 | 31 (6.47) | 8 (6.02) | 6 (5.61) | 7 (9.72) | 3 (3.75) | 7 (8.05) | 0.597 |
| Has diabetes history, n (%) | 119 (29.38) | 28 (34.57) | 26 (22.61) | 24 (31.58) | 19 (30.65) | 22 (30.99) | 0.419 | 150 (31.32) | 39 (29.32) | 36 (33.64) | 23 (31.94) | 21 (26.25) | 31 (35.63) | 0.692 |
| BMI at enrollment, mean (SD) | 28.24 (5.38) | 28.41 (5.25) | 28.25 (5.66) | 28.18 (5.49) | 28.83 (5.99) | 27.60 (4.35) | 0.766 | 29.80 (5.50) | 30.09 (5.39) | 30.11 (6.29) | 29.65 (5.06) | 29.30 (4.99) | 29.55 (5.50) | 0.815 |
| Smoking status, n (%) |  |  |  |  |  |  | 0.353 |  |  |  |  |  |  | 0.526 |
| Current | 113 (27.9) | 26 (32.1) | 35 (30.43) | 13 (17.11) | 19 (30.65) | 20 (28.17) |  | 182 (38.00) | 49 (36.84) | 38 (35.51) | 27 (37.5) | 32 (40) | 36 (41.38) |  |
| Former | 144 (35.56) | 30 (37.04) | 35 (30.43) | 32 (42.11) | 21 (33.87) | 26 (36.62) |  | 196 (40.92) | 60 (45.11) | 38 (35.51) | 32 (44.44) | 32 (40) | 34 (39.08) |  |
| Never | 143 (35.31) | 25 (30.86) | 42 (36.52) | 31 (40.79) | 22 (35.48) | 23 (32.39) |  | 98 (20.46) | 22 (16.54) | 31 (28.97) | 13 (18.06) | 16 (20) | 16 (18.39) |  |
| Missing | 5 (1.23) | 0.00 (0.00) | 3 (2.61) | 0 (0.00) | 0 (0.00) | 2 (2.82) |  | 3 (0.63) | 2 (1.5) | 0 (0) | 0 (0) | 0 (0) | 1 (1.15) |  |
| West African ancestry, mean (SD) | 0.76 (0.11) | 0.76 (0.12) | 0.77 (0.09) | 0.76 (0.11) | 0.76 (0.10) | 0.75 (0.12) | 0.908 | 0.72 (0.13) | 0.72 (0.12) | 0.73 (0.12) | 0.71 (0.14) | 0.72 (0.14) | 0.71 (0.15) | 0.725 |
| **Neighborhood Characteristics Percent Change, 1990 to 2006-2010** |  |  |  |  |  |  |  |  |  |  |  |  |  |  |
| Median household income, mean (SD) | .43 (37.66) | -20.96 (13.09) | -14.19 (14.03) | -3.00 (7.48) | 12.75 (12.79) | 41.43 (68.73) | **< 0.001** | 0.92  (25.36) | -18.34 (13.72) | -9.76 (12.94) | -0.86 (10.90) | 11.80 (10.35) | 34.95 (29.73) | **< 0.001** |
| Residents living below the federal poverty line, mean (SD) | -49.15 (118.06) | -196.39 (184.69) | -38.32 (45.48) | -22.82 (35.94) | 10.37 (38.46) | 21.13 (35.29) | **< 0.001** | -77.16  (168.09) | -247.38 (229.03) | -49.31 (58.54) | -26.58 (55.49) | 8.35 (47.48) | 28.32 (41.37) | **< 0.001** |
| College or more educated, mean (SD) | 43.23 (76.51) | -2.52 (26.87) | 21.23 (50.18) | 46.28 (40.04) | 48.10 (49.29) | 123.54 (123.44) | **< 0.001** | 34.69  (54.40) | 3.22 (25.09) | 16.22 (43.02) | 45.08 (46.91) | 44.88 (37.34) | 87.51 (71.67) | **< 0.001** |
| **Notes.** Neighborhood gentrification represents the sum of z scores of percent change of three socio-economic indicators (i.e., college or more educated adults aged 25 or more, number of residents  living below the federal poverty line, and median household income). The index was operationalized as five quintiles (very high, high, medium, low, and very low) based on the sample distribution. Sample for West African Ancestry: cases, n = 358, and controls, n = 458. Chi-square test for categorical variable and ANOVA test for continuous variables for total sample. | | | | | | | | | | | | | | |

**Supplementary Table 2. Descriptive characteristics of European American men in the NCI-Maryland Case-Control Study, by neighborhood gentrification quintiles**

|  | **European American cases, n = 364** | | | | | | | **European American controls, n = 544** | | | | | | |
| --- | --- | --- | --- | --- | --- | --- | --- | --- | --- | --- | --- | --- | --- | --- |
|  | Total | Very low | Low | Moderate | High | Very High | *P* value | Total | Very low | Low | Moderate | High | Very High | *P* value |
| **Individual-level Characteristics** |  | n = 55 | n = 47 | n = 65 | n = 105 | n = 92 |  |  | n = 91 | n = 89 | n = 149 | n = 107 | n = 108 |  |
| Age, years, mean (SD) | 64.02 (7.76) | 65.84 (8.08) | 64.94 (7.83) | 67.09 (7.68) | 64.99 (7.78) | 65.41 (8.31) | 0.505 | 66.86 (8.19) | 67.44 (8.67) | 66.80 (9.14) | 66.41 (7.77) | 66.20 (7.10) | 67.71 (8.56) |  |
| Education, n (%) |  |  |  |  |  |  | 0.313 |  |  |  |  |  |  | **0.036** |
| High school or less | 93 (25.55) | 12 (21.82) | 10 (21.28) | 15 (23.08) | 23 (21.9) | 33 (35.87) |  | 103 (18.93) | 19 (20.88) | 19 (21.35) | 18 (12.08) | 24 (22.43) | 23 (21.30) |  |
| Some college | 93 (25.55) | 11 (20.00) | 12 (25.53) | 15 (23.08) | 34 (32.38) | 21 (22.83) |  | 123 (22.61) | 20 (21.98) | 20 (22.47) | 28 (18.79) | 25 (23.36) | 30 (27.78) |  |
| College | 91 (25.00) | 17 (30.91) | 12 (25.53) | 17 (26.15) | 29 (27.62) | 16 (17.39) |  | 156 (28.68) | 23 (25.27) | 29 (32.58) | 42 (28.19) | 37 (34.58) | 25 (23.15) |  |
| Professional school | 87 (23.90) | 15 (27.27) | 13 (27.66) | 18 (27.69) | 19 (18.10) | 22 (23.91) |  | 162 (29.78) | 29 (31.87) | 21 (23.60) | 61 (40.94) | 21 (19.63) | 30 (27.78) |  |
| Missing | 0 (0) | 0 (0.00) | 0 (0.00) | 0 (0.00) | 0 (0.00) | 0 (0.00) |  |  | 0 (0.00) | 0 (0.00) | 0 (0.00) | 0 (0.00) 50 | 0 (0.00) |  |
| Income, n (%) |  |  |  |  |  |  | 0.617 |  |  |  |  |  |  | 0.150 |
| < $10,000 | 82 (22.53) | 7 (12.73) | 11 (23.40) | 15 (23.08) | 30 (28.57) | 19 (20.65) |  | 54 (9.93) | 10 (10.99) | 11 (12.36) | 15 (10.07) | 5 (4.67) | 13 (12.04) |  |
| $10,000-$30,000 | 72 (19.78) | 14 (25.45) | 6 (12.77) | 14 (21.54) | 16 (15.24) | 22 (23.91) |  | 127 (23.35) | 29 (31.87) | 18 (20.22) | 27 (18.12) | 29 (27.10) | 24 (22.22) |  |
| $30,000-$60,000 | 71 (19.51) | 8 (14.55) | 12 (25.53) | 15 (23.08) | 18 (17.14) | 18 (19.57) |  | 125 (22.98) | 18 (19.78) | 24 (26.97) | 30 (20.13) | 23 (21.50) | 30 (27.78) |  |
| $60,000-$90,000 | 116 (31.87) | 23 (41.82) | 14 (29.79) | 17 (26.15) | 35 (33.33) | 27 (29.35) |  | 206 (37.87) | 29 (31.87) | 27 (30.34) | 68 (45.64) | 45 (42.06) | 37 (34.26) |  |
| > $90,000 | 23 (6.32) | 3 (5.45) | 4 (8.51) | 4 (6.15) | 6 (5.71) | 6 (6.52) |  | 32 (5.88) | 5 (5.49) | 9 (10.11) | 9 (6.04) | 5 (4.67) | 4 (3.70) |  |
| Aspirin use, n (%) | 205 (56.32) | 30 (54.55) | 30 (63.83) | 34 (52.31) | 65 (61.90) | 46 (50.00) | 0.355 | 336 (61.76) | 57 (62.64) | 59 (66.29) | 93 (62.42) | 60 (56.07) | 67 (62.04) | 0.681 |
| Has family history of prostate cancer, n (%) | 46 (12.64) | 2 (3.64) | 5 (10.64) | 12 (18.46) | 12 (11.43) | 15 (16.30) | 0.113 | 40 (7.35) | 8 (8.79) | 7 (7.87) | 7 (4.7) | 8 (7.48) | 10 (9.26) | 0.657 |
| Has diabetes history, n (%) | 59 (16.21) | 11 (20.00) | 5 (10.64) | 10 (15.38) | 19 (18.10) | 14 (15.22) | 0.730 | 100 (18.38) | 14 (15.38) | 10 (11.24) | 36 (24.16) | 20 (18.69) | 20 (18.52) | 0.141 |
| BMI at enrollment, mean (SD) | 28.19 (4.90) | 28.13 (3.79) | 28.62 (5.41) | 27.93 (4.66) | 28.01 (4.16) | 28.18 (3.91) | 0.934 | 28.26 (5.04) | 28.10 (5.16) | 28.35 (4.41) | 28.62 (5.69) | 28.22 (4.79) | 27.87 (4.74) |  |
| Smoking status, n (%) |  |  |  |  |  |  | 0.654 |  |  |  |  |  |  | 0.822 |
| Current | 144 (39.56) | 24 (43.64) | 19 (40.43) | 26 (40.00) | 35 (33.33) | 40 (43.48) |  | 223 (40.99) | 36 (39.56) | 36 (40.45) | 66 (44.30) | 41 (38.32) | 44 (40.74) |  |
| Former | 163 (44.78) | 25 (45.45) | 22 (46.81) | 31 (47.69) | 45 (42.86) | 40 (43.48) |  | 262 (48.16) | 44 (48.35) | 44 (49.44) | 72 (48.32) | 53 (49.53) | 49 (45.37) |  |
| Never | 55 (15.11) | 6 (10.91) | 6 (12.77) | 8 (12.31) | 24 (22.86) | 11 (11.96) |  | 53 (9.74) | 10 (10.99) | 9 (10.11) | 8 (5.37) | 12 (11.21) | 14 (12.96) |  |
| Missing | 2 (0.55) | 0 (0.00) | 0 (0.00) | 0 (0.00) | 1 (0.95) | 1 (1.09) |  | 6 (1.1) | 1 (1.10) | 0 (0) | 3 (2.01) | 1 (0.93) | 1 (0.93) |  |
| West African ancestry, mean (SD) | 0.44 (0.35) | 0.06 (0.07) | 0.08 (0.08) | 0.07 (0.07) | 0.08 (0.10) | 0.08 (0.07) | 0.655 | .08 (0.09) | 0.08 (0.08) | 0.08 (0.06) | 0.07 (0.07) | .09(0.09) | 0.10 (0.12) | 0.055 |
| **Neighborhood Characteristics Percent Change, 1990 to 2006-2010** |  |  |  |  |  |  |  |  |  |  |  |  |  |  |
| Median household income, mean (SD) | 8.29 (22.97) | -11.33 (12.23) | -3.89 (12.88) | 4.12 (8.71) | 13.65 (9.83) | 32.25 (17.79) | **< 0.001** | 8.29 (22.97) | -13.60 (16.72) | -5.87 (13.83) | 2.53 (9.58) | 13.04 (8.26) | 33.78 (35.14) | **< 0.001** |
| Residents living below the federal poverty line, mean (SD) | -69.52 (153.59) | -304.33 (246.15) | -103.92 (92.25) | -50.31 (55.94) | -14.03 (56.45) | 22.49 (53.58) | **< 0.001** | -69.52 (153.59) | -298.10 (225.61) | -92.69 (96.14) | -35.15 (63.14) | -3.89 (48.90) | 20.37 (40.47) | **< 0.001** |
| College or more educated, mean (SD) | 53.04 (68.20) | 13.40 (24.50) | 33.36 (40.38) | 49.17 (31.55) | 53.08 (36.21) | 111.45 (123.52) | **< 0.001** | 53.04 (68.20) | 16.24 (22.37) | 31.95 (31.19) | 42.85 (33.40) | 50.27 (29.62) | 99.49 (110.33) | **< 0.001** |
| **Notes.** Neighborhood gentrification represents the sum of z scores of percent change of three socio-economic indicators (i.e., college or more educated adults aged 25 or more, number of residents  living below the federal poverty line, and median household income). The index was operationalized as five quintiles (very high, high, medium, low, and very low) based on the sample distribution. Sample for West African Ancestry: cases, n = 308, and controls, n = 509. Chi-square test for categorical variable and ANOVA test for continuous variables for total sample. | | | | | | | | | | | | | | |

**Supplementary Table 3. Association between neighborhood gentrification and National Comprehensive Cancer Network risk scores among African and European American men with prostate cancer**

|  | AA + EA men | | AA men | | EA men | | Low-income men | | High-income men | |
| --- | --- | --- | --- | --- | --- | --- | --- | --- | --- | --- |
| Gentrification (continuous) | Model 1, OR (95% CI) | Model 2, OR (95% CI) | Model 1, OR (95% CI) | Model 2, OR (95% CI) | Model 1, OR (95% CI) | Model 2, OR (95% CI) | Model 1, OR (95% CI) | Model 2,  OR (95% CI) | Model 1, OR (95% CI) | Model 2, OR (95% CI) |
| **NCCN Risk Scores** | | | | | | |  |  |  |  |
| Low | Ref. | Ref. | Ref. | Ref. | Ref. | Ref. | Ref. | Ref. | Ref. | Ref. |
| Intermediate | 0.95  [0.85, 1.05] | 0.94  [0.84, 1.05] | 0.93  [0.81, 1.07] | 0.91  [0.79, 1.04] | 0.99  [0.83, 1.19] | 1.02  [0.85, 1.22] | 1.03 [0.82,1.31] | 1.02  [0.81, 1.30] | 0.92  [0.81, 1.04] | 0.92 [0.81,1.04] |
| High | 0.95  [0.85, 1.07] | 0.96  [ 0.84, 1.08] | 0.93  [0.78, 1.12] | 0.95  [0.78, 1.16] | 1.01  [0.84, 1.22] | 1.02  [0.84, 1.23] | 1.08  [0.83, 1.40] | 1.08  [0.83, 1.41] | 0.91 [0.78,1.06] | 0.90 [0.77,1.06] |
| Regional/Metastatic | 0.94  [0.76, 1.16] | 0.92  [0.72, 1.17] | 0.97  [0.71, 1.32] | 1.01  [0.77, 1.31] | 0 .80  [0.55, 1.15] | 0.46  [0.20, 1.05] | 1.08  [0.70, 1.67] | 0.95  [0.59, 1.52] | 0.76 [0.52,1.11] | 0.76 [0.52,1.12] |

Notes. Abbreviations: AA, African American; EA, European American; RRR, Relative Risk Ratio; CI, Confidence Interval. Neighborhood deprivation index was constructed from a principal components analysis using 2000 census tract data for four dimensions of socioeconomic standing: education, employment, occupation, and poverty, standardized to have mean 0 and standard deviation of 1 and categorized to tertials. Neighborhood gentrification represents the sum of z scores of percent change of three socio-economic indicators (i.e.**,** college or more educated adults aged 25 or more, number of residents living below the federal poverty line, and median household income). The index was operationalized as continuous (higher scores indication greater gentrification. Model 1 adjusted for age at study entry (continuous), aspirin use (yes/no), family history of prostate cancer (first-degree relatives, yes/no), diabetes history (yes/no), body mass index at study entry (continuous), self-reported race (not included in race stratified analyses, African American, European American), smoking status (current, former, never, missing). Model 2 additionally adjusted for education (high school or less, some college, college, professional school, missing), individual income (< 30,000, $30,000–$60,000, $60,000–$90,000, >$90,000, missing). Low-income men defined as < $30,000 and middle/high income men as ≥ $30,000. Separate logistics regressions were performed comparing NCCN risk scores (i.e., intermediate, high, regional/metastatic) to low (AA + EA men intermediate models n = 554, high models n = 320, regional/metastatic models n = 185; AA men intermediate n =288, high models n = 153, regional/metastatic models n = 86; EA men intermediate n =266, high models n = 167, regional/metastatic models n = 99; Low-income men intermediate n =195, high models n = 113, regional/metastatic models n = 67; High-income men intermediate n = 359, high models n = 207, regional/metastatic models n = 118.

**Supplementary Table 4. Association of neighborhood gentrification with the National Comprehensive Cancer Network risk scores among African and European American men with prostate cancer, dichotomized as localized vs. regional or metastatic disease**

|  | AA + EA men | AA + EA men | AA men | | EA men | | Low-income men | | High-income men | |
| --- | --- | --- | --- | --- | --- | --- | --- | --- | --- | --- |
|  | Model 1 | Model 2 | Model 1 | Model 2 | Model 1 | Model 2 | Model 1 | Model 2 | Model 1 | Model 2 |
|  | n = 769 | n = 768 | n = 405 | n = 364 | n = 362 | n = 290 | n = 287 | n = 287 | n = 478 | n = 482 |
| Gentrification |  |  |  |  |  |  |  |  |  |  |
| Continuous score, OR [95% CI] |  |  |  |  |  |  |  |  |  |  |
| Localized (n = 729) | Ref. | Ref. | Ref. | Ref. | Ref. | Ref. | Ref. | Ref. | Ref. | Ref. |
| Regional or metastatic (n = 40) | 1.02 [0.85,1.24] | 1.00 [0.82,1.23] | 1.14 [0.94,1.38] | 1.15 [0.93,1.41] | 0.78 [0.56,1.08] | **0.61^*^ [0.40,0.94]** | 1.09  [0.83,1.44] | 1.05  [0.79,1.40] | 0.87  [0.62,1.21] | 0.88  [0.63,1.23] |
| Notes. Abbreviations: AA, African American; EA, European American, OR, Odds Ratio; CI, Confidence Interval.  Neighborhood gentrification represents the sum of z scores of percent change of three socio-economic indicators (i.e.**,** college or more educated adults aged 25 or more, number of residents living below the federal poverty line, and median household income). The index was operationalized as continuous with higher scores indicating greater gentrification. Disease stage was defined according to the National Comprehensive Cancer Network risk score categories with localized (low, intermediate, high/very high categories) vs. regional or metastatic. Model 1, logistic regression analysis adjusted for age at study entry (continuous), aspirin use (yes/no), family history of prostate cancer (first-degree relatives, yes/no), diabetes history (yes/no), body mass index at study entry (continuous), self-reported race (not included in stratified analyses, African American, European American), smoking status (current, former, never, missing). Model 2 additionally adjusted for education (high school or less, some college, college, professional school, missing), individual income (< $30,000, $30,000–$60,000, $60,000–$90,000, >$90,000, missing). Low-income men defined as < $30,000 and middle/high income men as ≥ $30,000 * *P* < 0.05. | | | | | | | | | | |

**Supplementary Table 5. Association between neighborhood gentrification and serum proteome signatures defining 3 biological pathways among African and European American men without prostate cancer (population controls), using MANOVA regression**

|  | AA + EA men | | AA men | | EA men | | Low-income men | | Middle/High income men | |
| --- | --- | --- | --- | --- | --- | --- | --- | --- | --- | --- |
| *Neighborhood Gentrification Operationalization* | Model 1, Beta (95% CI) | Model 2, Beta (95% CI) | Model 1, Beta (95% CI) | Model 2, Beta (95% CI) | Model 1, Beta (95% CI) | Model 2, Beta (95% CI) | Model 1, Beta (95% CI) | Model 2, Beta (95% CI) | Model 1, Beta (95% CI) | Model 2, Beta (95% CI) |
|  | N = 787 | N = 787 | n = 356 | n = 356 | n = 431 | n = 431 | n = 120 | n = 120 | n = 667 | n = 667 |
| **Suppression of Tumor Immunity** |  |  |  |  |  |  |  |  |  |  |
| Continuous score | -0.004 [-0.02,0.01] | -0.005 [-0.02,0.01] | -0.008 [-0.03,0.02] | -0.009 [-0.03,0.01] | -0.000 [-0.02,0.02] | -0.002 [-0.02,0.02] | 0.011 [-0.03,-0.06] | 0.009 [-0.04,0.06] | -0.007 [-0.02,0.01] | -0.009 [-0.03,0.01] |
| Quintiles |  |  |  |  |  |  |  |  |  |  |
| Q1 (very low) | Ref. | Ref. | Ref. | Ref. | Ref. | Ref. | Ref. | Ref. | Ref. | Ref. |
|  | -0.013 [-0.09,0.06] | -0.019 [-0.09,0.05] | 0.012 [-0.09,0.12] | -0.004 [-0.11,0.10] | -0.026 [-0.13,0.08] | -0.028 [-0.14,0.08] | -0.038 [-0.22,0.14] | -0.044 [-0.22,0.13] | -0.001 [-0.08,0.08] | -0.005 [-0.09,0.08] |
| Q3 | -0.076 [-0.15,0.00] | -0.075 [-0.15,0.00] | -0.042 [-0.16,0.08] | -0.045 [-0.17,0.08] | -0.092 [-0.19,0.00] | -0.080 [-0.17,0.01] | 0.026 [-0.18,0.23] | 0.027 [-0.18,0.23] | -0.085* [-0.16,-0.01] | -0.086* [-0.16,-0.01] |
| Q4 | 0.009 [-0.06,0.08] | 0.010 [-0.06,0.08] | 0.048 [-0.06,0.16] | 0.055 [-0.06,0.17] | -0.013 [-0.11,0.09] | -0.021 [-0.12,0.08] | -0.041 [-0.27,0.19] | -0.063 [-0.30,0.18] | 0.017 [-0.06,0.09] | 0.010 [-0.07,0.09] |
| Q5 (very high) | -0.055 [-0.13,0.02] | -0.057 [-0.13,0.02] | -0.089 [-0.20,0.02] | -0.094 [-0.21,0.02] | -0.018 [-0.12,0.08] | -0.019 [-0.12,0.08] | 0.004 [-0.20,0.21] | 0.004 [-0.21,0.21] | -0.068 [-0.15,0.01] | -0.076 [-0.16,0.00] |
| *P* trend | 0.225 | 0.222 | 0.275 | 0.276 | 0.776 | 0.720 | 0.993 | 0.960 | 0.170 | 0.112 |
| **Chemotaxis** |  |  |  |  |  |  |  |  |  |  |
| Continuous score | 0.003 [-0.01,0.02] | 0.000 [-0.02,0.02] | -0.008 [-0.03,0.02] | -0.011 [-0.03,0.01] | 0.014 [-0.01,0.04] | 0.010 [-0.01,0.03] | 0.013 [-0.03,0.06] | 0.013 [-0.03,0.06] | 0.000 [-0.02,0.02] | -0.003 [-0.02,0.01] |
| Quintiles |  |  |  |  |  |  |  |  |  |  |
| Q1 (very low) | Ref. | Ref. | Ref. | Ref. | Ref. | Ref. | Ref. | Ref. | Ref. | Ref. |
| Q2 | -0.010 [-0.09,0.07] | -0.017 [-0.10,0.06] | 0.015 [-0.09,0.12] | 0.004 [-0.10,0.11] | -0.027 [-0.14,0.09] | -0.032 [-0.15,0.08] | 0.045 [-0.13,0.22] | 0.036 [-0.14,0.21] | 0.021 [-0.07,0.11] | 0.016 [-0.07,0.10] |
| Q3 | -0.056 [-0.13,0.02] | -0.057 [-0.13,0.02] | -0.023 [-0.14,0.10] | -0.019 [-0.14,0.11] | -0.065 [-0.17,0.04] | -0.056 [-0.16,0.05] | 0.128 [-0.07,0.32] | 0.135 [-0.06,0.33] | -0.035 [-0.12,0.05] | -0.038 [-0.12,0.05] |
| Q4 | 0.034 [-0.04,0.11] | 0.029 [-0.05,0.11] | 0.077 [-0.04,0.19] | 0.077 [-0.04,0.19] | 0.007 [-0.10,0.11] | -0.004 [-0.11,0.10] | 0.003 [-0.22,0.23] | -0.016 [-0.25,0.21] | 0.047 [-0.04,0.13] | 0.040 [-0.04,0.12] |
| Q5 (very high) | -0.039 [-0.12,0.04] | -0.043 [-0.12,0.04] | -0.095 [-0.21,0.02] | -0.101 [-0.22,0.02] | 0.011 [-0.10,0.12] | 0.005 [-0.10,0.11] | 0.079 [-0.12,0.28] | 0.087 [-0.12,0.29] | -0.026 [-0.11,0.06] | -0.037 [-0.12,0.05] |
| *P* trend | 0.885 | 0.957 | 0.398 | 0.306 | 0.225 | 0.301 | 0.420 | 0.417 | 0.788 | 0.601 |
| **Inflammation** |  |  |  |  |  |  |  |  |  |  |
| Continuous score | -0.000 [-0.02,0.02] | -0.002 [-0.02,0.01] | -0.008 [-0.03,0.02] | -0.010 [-0.03,0.01] | 0.008 [-0.01,0.03] | 0.005 [-0.02,0.03] | 0.006 [-0.04,0.05] | 0.007 [-0.04,0.05] | -0.002 [-0.02,0.02] | -0.005 [-0.02,0.01] |
| Quintiles |  |  |  |  |  |  |  |  |  |  |
| Q1 (very low) | Ref. | Ref. | Ref. | Ref. | Ref. | Ref. | Ref. | Ref. | Ref. | Ref. |
| Q2 | -0.010 [-0.09,0.07] | -0.017 [-0.10,0.06] | 0.015 [-0.09,0.12] | 0.004 [-0.10,0.11] | -0.027 [-0.14,0.09] | -0.032 [-0.15,0.08] | -0.041 [-0.22,0.14] | -0.051 [-0.23,0.13] | 0.006 [-0.08,0.09] | 0.002 [-0.09,0.09] |
| Q3 | -0.056 [-0.13,0.02] | -0.057 [-0.13,0.02] | -0.023 [-0.14,0.10] | -0.019 [-0.14,0.11] | -0.065 [-0.17,0.04] | -0.056 [-0.16,0.05] | 0.056 [-0.15,0.26] | 0.065 [-0.14,0.27] | -0.065 [-0.15,0.02] | -0.067 [-0.15,0.02] |
| Q4 | 0.034 [-0.04,0.11] | 0.029 [-0.05,0.11] | 0.077 [-0.04,0.19] | 0.077 [-0.04,0.19] | 0.007 [-0.10,0.11] | -0.004 [-0.11,0.10] | -0.042 [-0.28,0.19] | -0.062 [-0.30,0.18] | 0.043 [-0.04,0.13] | 0.037 [-0.05,0.12] |
| Q5 (very high) | -0.039 [-0.12,0.04] | -0.043 [-0.12,0.04] | -0.095 [-0.21,0.02] | -0.101 [-0.22,0.02] | 0.011 [-0.10,0.12] | 0.005 [-0.10,0.11] | 0.016 [-0.19,0.22] | 0.025 [-0.19,0.24] | -0.052 [-0.14,0.03] | -0.061 [-0.15,0.02] |
| *P* trend | 0.573 | 0.502 | 0.349 | 0.315 | 0.722 | 0.839 | 0.883 | 0.862 | 0.446 | 0.326 |

Notes. Abbreviations: AA, African American; EA, European American; OR, odds ratio; CI, confidence interval. Neighborhood gentrification represents the sum of z scores of percent change of three socio-economic indicators (i.e.**,** college or more educated adults aged 25 or more, number of residents living below the federal poverty line, and median household income). The index was operationalized as continuous (higher scores indicate greater gentrification) and quintiles. Model 1 adjusted for age at study entry (continuous), aspirin use (yes/no), family history of prostate cancer (first-degree relatives, yes/no), diabetes history (yes/no), body mass index at study entry (continuous), self-reported race (not included in race stratified analyses, African American, European American), West African ancestry, smoking status (current, former, never, missing). Model 2 additionally adjusted for education (high school or less, some college, college, professional school, missing), individual income (not included in income stratified analyses, < $30,000, $30,000–$60,000, $60,000–$90,000, >$90,000, missing). Low-income men defined as < $30,000 and middle/high income men as ≥ $30,000. Statistical significance was determined with a Bonferroni adjusted *P* < 0.0004 based on an alpha of 0.05 for 108 tests.

**Supplementary Table 6. MANOVA Regression Model Statistics for the association between neighborhood gentrification and serum proteome signatures defining 3 biological pathways among African and European American men without prostate cancer (population controls)**

| **AA + EA men (MANOVA)** | | | | | | | | | | |  |
| --- | --- | --- | --- | --- | --- | --- | --- | --- | --- | --- | --- |
| **Model 1 (N = 787)** | | | | | | **Model 2 (N= 787)** | | | | |  |
| **Continuous Model** |  |  | |  |  |  |  | |  |  |  |
|  | **Model**  **Statistics** | **F (df1, df2)** | | **F statistic** | ***P* value** | **Model Statistics** | **F (df1, df2)** | | **F statistic** | ***P* value** |  |
|  | | | | | | | | | | | |
| Wilk's lambda | 0.60 | 33.00, 2278.10 | | 13.23 | <0.001 | 0.56 | 57.00, 2281.80 | | 8.55 | <0.001 |  |
| Pillai's trace | 0.44 | 33.00, 2325.00 | | 12.25 | <0.001 | 0.50 | 57.00, 2301.00 | | 8.04 | <0.001 |  |
| Lawley-Hotelling trace | 0.61 | 33.00, 2315.00 | | 14.24 | <0.001 | 0.68 | 57.00, 2291.00 | | 9.08 | <0.001 |  |
| Roy's largest Root | 0.47 | 11.00,775.00 | | 33.32 | <0.001 | 0.48 | 19.00, 767.00 | | 19.53 | <0.001 |  |
|  |  |  | |  |  |  |  | |  |  |  |
| **Pathways** | **R squared** | **RMSE** | | **F statistic** | ***P* value** | **R squared** | **RMSE** | | **F statistic** | ***P* value** |  |
| Suppression | 0.11 | 0.33 | | 8.28 | <0.001 | 0.12 | 0.33 | | 5.68 | <0.001 |  |
| Chemotaxis | 0.11 | 0.35 | | 8.75 | <0.001 | 0.13 | 0.35 | | 6.07 | <0.001 |  |
| Inflammation | 0.05 | 0.35 | | 3.93 | <0.001 | 0.07 | 0.35 | | 3.04 | <0.001 |  |
|  |  |  | |  |  |  |  | |  |  |  |
| **Quintiles Model** |  |  | |  |  |  |  | |  |  |  |
|  | **Model Statistics** | **F (df1, df2)** | | **F statistic** | ***P* value** | **Model Statistics** | **F (df1, df2)** | | **F statistic** | ***P* value** |  |
| Wilk's lambda | 0.59 | 42.00, 2285.00 | | 10.76 | <0.001 | 0.55 | 66.00, 2276.40 | | 7.60 | <0.001 | |
| Pillai's trace | 0.46 | 42.00, 2316.00 | | 10.01 | <0.001 | 0.51 | 66.00, 2292.00 | | 7.17 | <0.001 | |
| Lawley-Hotelling trace | 0.63 | 42.00, 2306.00 | | 11.54 | <0.001 | 0.70 | 66.00, 2282.00 | | 8.05 | <0.001 | |
| Roy's largest Root | 0.48 | 14.00, 772.00 | | 26.31 | <0.001 | 0.49 | 22.00, 764.00 | | 16.92 | <0.001 | |
|  |  |  | |  |  |  |  | |  |  |  |
| **Pathways** | **R squared** | **RMSE** | | **F statistic** | ***P* value** | **R squared** | **RMSE** | | **F statistic** | ***P* value** |  |
| Suppression | 0.11 | 0.33 | | 7.08 | <0.001 | 0.13 | 0.33 | | 5.25 | <0.001 |  |
| Chemotaxis | 0.11 | 0.35 | | 7.09 | <0.001 | 0.13 | 0.35 | | 5.35 | <0.001 |  |
| Inflammation | 0.06 | 0.35 | | 3.54 | <0.001 | 0.08 | 0.35 | | 2.89 | <0.001 |  |
|  | | | |  | | |  | | | |  |
| **AA men (MANOVA)** | | | | | | | | | | |  |
| **Model 1 (N = 356)** | | | | | | **Model 2 (N = 356)** | | | | |  |
| **Continuous Model** |  |  | |  |  |  |  | |  |  | |
|  | **Model Statistics** | **F (df1, df2)** | | **F statistic** | ***P* value** | **Model Statistics** | **F (df1, df2)** | | **F statistic** | ***P* value** | |
| Wilk's lambda | 0.75 | 30.00, 1007.40 | | 3.42 | <0.001 | 0.68 | 54.00, 999.00 | | 2.58 | <0.001 | |
| Pillai's trace | 0.27 | 30.00, 1035.00 | | 3.41 | <0.001 | 0.36 | 54.00, 1011.00 | | 2.58 | <0.001 | |
| Lawley-Hotelling trace | 0.30 | 30.00, 1025.00 | | 3.42 | <0.001 | 0.42 | 54.00, 1001.00 | | 2.57 | <0.001 | |
| Roy's largest Root | 0.13 | 10.00, 345.00 | | 4.65 | <0.001 | 0.18 | 18.00, 337.00 | | 3.37 | <0.001 | |
|  |  |  | |  |  |  |  | |  |  |  |
| **Pathways** | **R squared** | **RMSE** | | **F statistic** | ***P* value** | **R squared** | **RMSE** | | **F statistic** | ***P* value** |  |
| Suppression | 0.12 | 0.35 | | 4.53 | <0.001 | 0.15 | 0.35 | | 3.22 | <0.001 |  |
| Chemotaxis | 0.09 | 0.35 | | 3.26 | 0.001 | 0.12 | 0.35 | | 2.57 | 0.001 |  |
| Inflammation | 0.10 | 0.36 | | 3.81 | <0.001 | 0.13 | 0.36 | | 2.88 | <0.001 |  |
|  |  |  | |  |  |  |  | |  |  |  |
| **Quintiles Model** |  |  | |  |  |  |  | |  |  |  |
|  | **Model Statistics** | **F (df1, df2)** | | **F statistic** | ***P* value** | **Model Statistics** | **F (df1, df2)** | | **F statistic** | ***P* value** |  |
| Wilk's lambda | 0.73 | 39.00, 1007.60 | | 2.92 | <0.001 | 0.66 | 63.00, 991.90 | | 2.39 | <0.001 |  |
| Pillai's trace | 0.30 | 39.00, 1026.00 | | 2.91 | <0.001 | 0.39 | 63.00, 1002.00 | | 2.40 | <0.001 |  |
| Lawley-Hotelling trace | 0.34 | 39.00, 1016.00 | | 2.92 | <0.001 | 0.46 | 63.00, 992.00 | | 2.39 | <0.001 |  |
| Roy's largest Root | 0.15 | 13.00, 342.00 | | 3.94 | <0.001 | 0.20 | 21.00, 334.00 | | 3.14 | <0.001 |  |
|  |  |  | |  |  |  |  | |  |  |  |
| **Pathways** | **R squared** | **RMSE** | | **F statistic** | ***P* value** | **R squared** | **RMSE** | | **F statistic** | ***P* value** |  |
| Suppression | 0.13 | 0.35 | | 3.91 | <0.001 | 0.16 | 0.35 | | 3.06 | <0.001 |  |
| Chemotaxis | 0.11 | 0.35 | | 3.09 | <0.001 | 0.14 | 0.35 | | 2.55 | <0.001 |  |
| Inflammation | 0.12 | 0.36 | | 3.50 | <0.001 | 0.15 | 0.36 | | 2.84 | <0.001 |  |
|  |  |  | |  |  |  |  | |  |  |  |
| **EA men (MANOVA)** | | | | | | | | | | |  |
| **Model 1 (N = 431)** | | | | | | **Model 2 (N = 431)** | | | | |  |
| **Continuous Model** |  |  | |  |  |  |  | |  |  |  |
|  | **Model Statistics** | **F (df1, df2)** | | **F statistic** | ***P* value** | **Model Statistics** | **F (df1, df2)** | | **F statistic** | ***P* value** |  |
| Wilk's lambda | 0.75 | 30.00, 1227.60 | | 4.27 | <0.001 | 0.70 | 51.00, 1224.40 | | 3.01 | <0.001 | |
| Pillai's trace | 0.27 | 30.00, 1260.00 | | 4.18 | <0.001 | 0.32 | 51.00, 1239.00 | | 2.94 | <0.001 | |
| Lawley-Hotelling trace | 0.31 | 30.00, 1250.00 | | 4.35 | <0.001 | 0.38 | 51.00, 1229.00 | | 3.07 | <0.001 | |
| Roy's largest Root | 0.19 | 10.00, 420.00 | | 8.09 | <0.001 | 0.23 | 17.00, 413.00 | | 5.70 | <0.001 | |
|  |  |  | |  |  |  |  | |  |  |  |
| **Pathways** | **R squared** | **RMSE** | | **F statistic** | ***P* value** | **R squared** | **RMSE** | | **F statistic** | ***P* value** |  |
| Suppression | 0.10 | 0.32 | | 4.49 | <0.001 | 0.12 | 0.32 | | 3.21 | 0.0000 |  |
| Chemotaxis | 0.06 | 0.35 | | 2.79 | 0.0024 | 0.08 | 0.35 | | 2.22 | 0.0037 |  |
| Inflammation | 0.05 | 0.34 | | 2.01 | 0.0306 | 0.06 | 0.34 | | 1.58 | 0.0656 |  |
|  |  |  | |  |  |  |  | |  |  |  |
| **Quintiles Model** |  |  | |  |  |  |  | |  |  |  |
|  | **Model Statistics** | **F (df1, df2)** | | **F statistic** | ***P* value** | **Model Statistics** | **F (df1, df2)** | | **F statistic** | ***P* value** |  |
| Wilk's lambda | 0.73 | 39.00, 1229.70 | | 3.54 | <0.001 | 0.69 | 60.00, 1218.10 | | 2.69 | <0.001 |  |
| Pillai's trace | 0.29 | 39.00, 1251.00 | | 3.47 | <0.001 | 0.34 | 60.00, 1230.00 | | 2.63 | <0.001 |  |
| Lawley-Hotelling trace | 0.34 | 39.00, 1241.00 | | 3.61 | <0.001 | 0.41 | 60.00, 1220.00 | | 2.75 | <0.001 |  |
| Roy's largest Root | 0.21 | 13.00, 417.00 | | 6.61 | <0.001 | 0.24 | 20.00, 410.00 | | 4.99 | <0.001 |  |
|  |  |  | |  |  |  |  | |  |  |  |
| **Pathways** | **R squared** | **RMSE** | | **F statistic** | ***P* value** | **R squared** | **RMSE** | | **F statistic** | ***P* value** |  |
| Suppression | 0.11 | 0.32 | | 3.88 | <0.001 | 0.12 | 0.32 | | 2.90 | <0.001 |  |
| Chemotaxis | 0.07 | 0.35 | | 2.29 | 0.006 | 0.09 | 0.35 | | 1.95 | 0.009 |  |
| Inflammation | 0.05 | 0.34 | | 1.77 | 0.046 | 0.07 | 0.35 | | 1.44 | 0.101 |  |
|  |  |  | |  |  |  |  | |  |  |  |
| **Low-income men (MANOVA)** | | | | | | | | | | |  |
| **Model 1 (N = 120)** | | | | | | **Model 2 (N= 120)** | | | | |  |
| **Continuous Model** |  |  | |  |  |  |  | |  |  |  |
|  | **Model**  **Statistics** | **F (df1, df2)** | | **F statistic** | ***P* value** | **Model Statistics** | **F (df1, df2)** | | **F statistic** | ***P* value** |  |
|  | | | | | | | | | | | |
| Wilk's lambda | 0.62 | 33.00, 313.00 | | 1.66 | 0.016 | 0.60 | 42.00 | 306.30 | 1.38 | 0.066 |  |
| Pillai's trace | 0.43 | 33.00, 324.00 | | 1.66 | 0.016 | 0.47 | 42.00 | 315.00 | 1.39 | 0.064 |  |
| Lawley-Hotelling trace | 0.52 | 33.00, 314.00 | | 1.66 | 0.016 | 0.57 | 42.00 | 305.00 | 1.38 | 0.068 |  |
| Roy's largest Root | 0.27 | 11.00, 108.00 | | 2.66 | 0.005 | 0.29 | 14.00 | 105.00 | 2.14 | 0.015 |  |
|  |  |  | |  |  |  |  | |  |  |  |
| **Pathways** | **R squared** | **RMSE** | | **F statistic** | **P value** | **R squared** | **RMSE** | | **F statistic** | **P value** |  |
| Suppression | 0.17 | 0.35 | | 2.00 | 0.035 | 0.18 | 0.35 | | 1.64 | 0.080 |  |
| Chemotaxis | 0.17 | 0.34 | | 1.96 | 0.039 | 0.18 | 0.34 | | 1.66 | 0.076 |  |
| Inflammation | 0.15 | 0.35 | | 1.79 | 0.064 | 0.17 | 0.35 | | 1.52 | 0.117 |  |
|  |  |  | |  |  |  |  | |  |  |  |
| **Quintiles Model** |  |  | |  |  |  |  | |  |  |  |
|  | **Model Statistics** | **F (df1, df2)** | | **F statistic** | ***P* value** | **Model Statistics** | **F (df1, df2)** | | **F statistic** | ***P* value** |  |
| Wilk's lambda | 0.59 | 42.00, 306.30 | | 1.44 | 0.044 | 0.56 | 51.00 | 298.50 | 1.27 | 0.113 | |
| Pillai's trace | 0.48 | 42.00, 315.00 | | 1.45 | 0.043 | 0.53 | 51.00 | 306.00 | 1.28 | 0.107 | |
| Lawley-Hotelling trace | 0.59 | 42.00, 305.00 | | 1.44 | 0.045 | 0.65 | 51.00 | 296.00 | 1.27 | 0.119 | |
| Roy's largest Root | 0.30 | 14.00, 105.00 | | 2.25 | 0.010 | 0.31 | 17.00 | 102.00 | 1.88 | 0.029 | |
|  |  |  | |  |  |  |  | |  |  |  |
| **Pathways** | **R squared** | **RMSE** | | **F statistic** | **P value** | **R squared** | **RMSE** | | **F statistic** | **P value** |  |
| Suppression | 0.17 | 0.35 | | 1.56 | 0.102 | 0.19 | 0.35 | | 1.36 | 0.171 |  |
| Chemotaxis | 0.18 | 0.34 | | 1.64 | 0.079 | 0.20 | 0.34 | | 1.48 | 0.119 |  |
| Inflammation | 0.16 | 0.35 | | 1.45 | 0.142 | 0.18 | 0.36 | | 1.32 | 0.195 |  |
|  | | | |  | | |  | | | |  |
| **Middle/High income men (MANOVA)** | | | | | | | | | | |  |
| **Model 1 (N = 667)** | | | | | | **Model 2 (N = 667)** | | | | |  |
| **Continuous Model** |  |  | |  |  |  |  | |  |  | |
|  | **Model Statistics** | **F (df1, df2)** | | **F statistic** | **P value** | **Model Statistics** | **F (df1, df2)** | | **F statistic** | **P value** | |
| Wilk's lambda | 0.58 | 33.00 | 1924.60 | 12.02 | <0.001 | 0.57 | 45.00 | 1928.80 | 9.01 | <0.001 | |
| Pillai's trace | 0.47 | 33.00 | 1965.00 | 10.99 | <0.001 | 0.48 | 45.00 | 1953.00 | 8.26 | <0.001 | |
| Lawley-Hotelling trace | 0.66 | 33.00 | 1955.00 | 13.09 | <0.001 | 0.68 | 45.00 | 1943.00 | 9.80 | <0.001 | |
| Roy's largest Root | 0.53 | 11.00 | 655.00 | 31.48 | <0.001 | 0.54 | 15.00 | 651.00 | 23.25 | <0.001 | |
|  |  |  | |  |  |  |  | |  |  |  |
| **Pathways** | **R squared** | **RMSE** | | **F statistic** | **P value** | **R squared** | **RMSE** | | **F statistic** | **P value** |  |
| Suppression | 0.10 | 0.33 | | 6.60 | <0.001 | 0.11 | 0.33 | | 5.28 | 0.00 |  |
| Chemotaxis | 0.11 | 0.36 | | 7.27 | <0.001 | 0.12 | 0.35 | | 5.79 | 0.00 |  |
| Inflammation | 0.05 | 0.35 | | 3.00 | <0.001 | 0.06 | 0.35 | | 2.58 | 0.00 |  |
|  |  |  | |  |  |  |  | |  |  |  |
| **Quintiles Model** |  |  | |  |  |  |  | |  |  |  |
|  | **Model Statistics** | **F (df1, df2)** | | **F statistic** | **P value** | **Model Statistics** | **F (df1, df2)** | | **F statistic** | **P value** |  |
| Wilk's lambda | 0.56 | 42.00 | 1929.00 | 9.79 | <.001 | 0.56 | 54.00 | 1925.60 | 7.76 | <.001 |  |
| Pillai's trace | 0.49 | 42.00 | 1956.00 | 9.01 | <.001 | 0.50 | 54.00 | 1944.00 | 7.16 | <.001 |  |
| Lawley-Hotelling trace | 0.69 | 42.00 | 1946.00 | 10.61 | <.001 | 0.70 | 54.00 | 1934.00 | 8.39 | <.001 |  |
| Roy's largest Root | 0.53 | 14.00 | 652.00 | 24.71 | <.001 | 0.54 | 18.00 | 648.00 | 19.34 | <.001 |  |
|  |  |  | |  |  |  |  | |  |  |  |
| **Pathways** | **R squared** | **RMSE** | | **F statistic** | **P value** | **R squared** | **RMSE** | | **F statistic** | **P value** |  |
| Suppression | 0.11 | 0.33 | | 5.95 | <.001 | 0.12 | 0.33 | | 4.94 | <.001 |  |
| Chemotaxis | 0.12 | 0.35 | | 6.08 | <.001 | 0.12 | 0.35 | | 5.10 | <.001 |  |
| Inflammation | 0.06 | 0.35 | | 2.99 | <.001 | 0.07 | 0.35 | | 2.62 | <.001 |  |

Notes. Abbreviations: AA, African American; EA, European American; OR, odds ratio; CI, confidence interval. Neighborhood gentrification represents the sum of z scores of percent change of three socio-economic indicators (i.e.**,** college or more educated adults aged 25 or more, number of residents living below the federal poverty line, and median household income). index was operationalized as continuous (higher scores indicate greater gentrification) and quintiles. Model 1 adjusted for age at study entry (continuous), aspirin use (yes/no), family history of prostate cancer (first-degree relatives, yes/no), diabetes history (yes/no), body mass index at study entry (continuous), self-reported race (not included in stratified race analyses, African American, European American), West African ancestry, smoking status (current, former, never, missing). Model 2 additionally adjusted for education (high school or less, some college, college, professional school, missing), individual income (not included in income stratified analyses, < $30,000, $30,000–$60,000, $60,000–$90,000, >$90,000, missing). Low-income men defined as < $30,000 and middle/high income men as ≥ $30,000. Statistical significance was determined with a Bonferroni adjusted P < 0.0004 based on an alpha of 0.05 for 108 tests.

**Supplementary Table 7. Association between neighborhood gentrification and all-cause and disease-specific mortality among African and European American men with prostate cancer**

|  | AA + EA men, n = 769 | | AA men, n = 405 | | EA men, n = 364 | | Low-income men, n = 287 | | High-income men, n = 482 | |
| --- | --- | --- | --- | --- | --- | --- | --- | --- | --- | --- |
| Median survival time, years | 5.72 | | 4.86 | | 6.14 | | 4.87 | | 6.13 | |
|  | Model 1 | Model 2 | Model 1 | Model 2 | Model 1 | Model 2 | Model 1 | Model 2 | Model 1 | Model 2 |
| **All-cause mortality** HR (95% CI) | 1.07 [0.99-1.16] | 1.06 [0.98-1.16] | 1.06 [0.95-1.18] | 1.04 [0.94-1.16] | 1.11 [0.96-1.28] | 1.12 [0.97-1.29] | 1.07 [0.94,1.21] | 1.09 [0.95,1.24] | 1.00 [0.89,1.13] | 1.00 [0.89,1.12] |
| Quintiles |  |  |  |  |  |  |  |  |  |  |
| Q1 (very low) |  |  |  |  |  |  |  |  |  |  |
| Q2 | 1.16 [0.73,1.87] | 1.16 [0.72,1.87] | 1.09 [0.61,1.94] | 1.18 [0.65,2.15] | 1.22 [0.51,2.95] | 1.47 [0.59,3.69] | 1.16 [0.53,2.53] | 1.12 [0.51,2.46] | 1.25 [0.66,2.35] | 1.19 [0.62,2.26] |
| Q3 | 1.17 [0.73,1.87] | 1.12 [0.70,1.81] | 1.17 [0.63,2.18] | 1.24 [0.65,2.34] | 1.16 [0.53,2.53] | 1.39 [0.62,3.12] | 0.95 [0.42,2.15] | 0.81 [0.35,1.87] | 1.20 [0.64,2.25] | 1.17 [0.63,2.19] |
| Q4 | 1.51 [0.97-2.36] | 1.43 [0.91,2.25] | 1.27 [0.68,2.36] | 1.28 [0.68,2.43] | 1.83 [0.90,3.75] | 2.11 [1.00,4.49] | 1.52 [0.70,3.31] | 1.50 [0.69,3.25] | 1.21 [0.66,2.23] | 1.10 [0.59,2.06] |
| Q5 (very high) | 1.27 [0.80,2.00] | 1.22 [0.77,1.95] | 1.16 [0.63,2.14] | 1.17 [0.63,2.20] | 1.43 [0.68,3.01] | 1.71 [0.79,3.70] | 1.25 [0.58,2.66] | 1.28 [0.61,2.72] | 1.03 [0.55,1.90] | 1.03 [0.55,1.92] |
| *p* for trend | 0.196 | 0.290 |  |  |  |  |  |  |  |  |
| **Prostate cancer-specific mortality** |  |  |  |  |  |  |  |  |  |  |
| Cox Proportional Hazard Regression, CSHR (95% CI) | 1.12 [0.95,1.33] | 1.14 [0.96,1.34] | -- | -- | -- | -- | -- | -- | -- | -- |
| Fine and Gray Regression, SHR (95% CI) | 1.11 [0.97,1.27] | 1.13 [0.99,1.28] | -- | -- | -- | -- | -- | -- | -- | -- |

Notes. Abbreviations: AA, African American; EA, European American; HR, Hazard Ratio; SHR, subdistribution hazard ratio; CSHR, cause-specific hazard ratio; CI, Confidence Interval. Exponentiated coefficients; 95% confidence intervals in brackets. Neighborhood gentrification represents the sum of z scores of percent change of three socio-economic indicators (i.e.**,** college or more educated adults aged 25 or more, number of residents living below the federal poverty line, and median household income). The index was operationalized as continuous (higher scores indicate greater gentrification) and quintiles. Model 1, Cox regression analysis adjusted for age at study entry (continuous), aspirin use (yes/no), family history of prostate cancer (first-degree relatives, yes/no), diabetes history (yes/no), body mass index at study entry (continuous), self-reported race (not included in race stratified analyses, African American, European American), smoking status (current, former, never, missing), treatment (none, surgery, radiation, hormone, combination, missing), National Comprehensive Cancer Network risk score categories (low, intermediate, high/very high, regional/metastatic). Model 2 additionally adjusted for education (high school or less, some college, college, professional school, missing), individual income (not included in income stratified analyses, < $30,000, $30,000–$60,000, $60,000–$90,000, >$90,000, missing). Low-income men defined as < $30,000 and middle/high income men as ≥ $30,000.

**Supplementary Figure 1.** MANOVA regression for the association of neighborhood gentrification with the suppression pathway activity scores among African (AA) and European American (EA) men without prostate cancer (population controls). Beta coefficients (β) and 95% confidence interval (CI) linking neighborhood gentrification to suppression scores with gentrification coded as **A)** continuous score and **B)** quintiles, stratified by self-reported race/ethnicity. Model 1 adjusted for all baseline covariates, whereas Model 2 is adjusted for all covariates with the additional inclusion of education and individual income.

**Supplementary Figure 2.** MANOVA regression for the association of neighborhood gentrification with the chemotaxis pathway activity scores among African (AA) and European American (EA) men without prostate cancer (population controls). Beta coefficients (β) and 95% confidence interval (CI) linking neighborhood gentrification to chemotaxis scores with gentrification coded as **A)** continuous score and **B)** quintiles, stratified by self-reported race/ethnicity. Model 1 adjusted for all baseline covariates, whereas Model 2 is adjusted for all covariates with the additional inclusion of education and individual income.

**Supplementary Figure 3.** MANOVA regression for the association of neighborhood gentrification with the inflammation pathway activity scores among African (AA) and European American (EA) men without a prostate cancer diagnosis (population controls). Beta coefficients (β) and 95% confidence interval (CI) linking neighborhood gentrification to inflammation scores with gentrification coded as **A)** continuous score and **B)** quintiles, stratified by self-reported race/ethnicity. Model 1 adjusted for all baseline covariates, whereas Model 2 is adjusted for all covariates with the additional inclusion of education and individual income.

**Supplementary Figure 4.** Association of neighborhood gentrification with all-cause and prostate cancer-specific mortality among African (AA) and European American (EA) men with prostate cancer. Hazard ratios (HR) and 95% confidence interval (CI) for the association of neighborhood gentrification with all-cause mortality, coded as **A)** continuous score and **B)** quintiles, stratified by self-reported race/ethnicity. **C**) Prostate cancer-specific hazard ratios (CSHR) and subdistribution hazard ratios (SHR) using Cox and Fine & Gray regression models, respectively, with neighborhood gentrification as continuous score. Model 1 adjusted for all baseline covariates, whereas Model 2 is adjusted for all covariates with the additional inclusion of education and individual income.
